# A Non-canonical RNA Silencing Pathway Promotes mRNA Degradation in Basal Fungi

**DOI:** 10.1371/journal.pgen.1005168

**Published:** 2015-04-13

**Authors:** Trung Anh Trieu, Silvia Calo, Francisco E. Nicolás, Ana Vila, Simon Moxon, Tamas Dalmay, Santiago Torres-Martínez, Victoriano Garre, Rosa M. Ruiz-Vázquez

**Affiliations:** 1 Department of Genetics and Microbiology, University of Murcia, Murcia, Spain; 2 The Genome Analysis Centre, University of East Anglia, Norwich, United Kingdom; 3 School of Biological Sciences, University of East Anglia, Norwich, United Kingdom; University College Dublin, IRELAND

## Abstract

The increasing knowledge on the functional relevance of endogenous small RNAs (esRNAs) as riboregulators has stimulated the identification and characterization of these molecules in numerous eukaryotes. In the basal fungus *Mucor circinelloides*, an emerging opportunistic human pathogen, esRNAs that regulate the expression of many protein coding genes have been described. These esRNAs share common machinery for their biogenesis consisting of an RNase III endonuclease Dicer, a single Argonaute protein and two RNA-dependent RNA polymerases. We show in this study that, besides participating in this canonical *dicer*-dependent RNA interference (RNAi) pathway, the *rdrp* genes are involved in a novel *dicer*-independent degradation process of endogenous mRNAs. The analysis of esRNAs accumulated in wild type and silencing mutants demonstrates that this new *rdrp*-dependent *dicer*-independent regulatory pathway, which does not produce sRNA molecules of discrete sizes, controls the expression of target genes promoting the specific degradation of mRNAs by a previously unknown RNase. This pathway mainly regulates conserved genes involved in metabolism and cellular processes and signaling, such as those required for heme biosynthesis, and controls responses to specific environmental signals. Searching the *Mucor* genome for candidate RNases to participate in this pathway, and functional analysis of the corresponding knockout mutants, identified a new protein, R3B2. This RNase III-like protein presents unique domain architecture, it is specifically found in basal fungi and, besides its relevant role in the *rdrp*-dependent *dicer*-independent pathway, it is also involved in the canonical dicer-dependent RNAi pathway, highlighting its crucial role in the biogenesis and function of regulatory esRNAs. The involvement of RdRPs in RNA degradation could represent the first evolutionary step towards the development of an RNAi mechanism and constitutes a genetic link between mRNA degradation and post-transcriptional gene silencing.

## Author Summary

Most eukaryotic organisms produce different classes of endogenous small RNA (esRNA) molecules that suppress gene expression through RNA interference (RNAi) pathways. These pathways, which may differ among organisms, are normally involved in genome defense, heterochromatin formation and regulation of genes involved in multiple cellular functions. In the basal fungus *Mucor circinelloides*, an opportunistic human pathogen, we previously demonstrated that biogenesis of a large group of esRNA molecules requires a basic RNAi machinery consisting of a Dicer-like protein, an Argonaute nuclease and two RNA-dependent RNA polymerases. This canonical *dicer*-dependent pathway regulates different cellular processes, such as vegetative sporulation. Besides those esRNAs generated by this canonical RNAi pathway, we have identified a new *rdrp*-dependent *dicer*-independent esRNA class. These esRNAs are produced by a degradation pathway in which the RdRP proteins signal specific transcripts that will be degraded by a newly identified RNase. This RNase, named R3B2, presents unique domain architecture, can only be found in basal fungi and it is also involved in the canonical *dicer*-dependent RNAi pathway. Our results expand the role of RdRPs in gene silencing and reveal the involvement of these proteins in a new RNA degradation process that could represent the first step in the evolution of RNAi.

## Introduction

Since the discovery of RNAi in *Caenorhabditis elegans* [1], our knowledge on the crucial role of endogenous small RNA (esRNA) as riboregulators has increased dramatically. Multiple classes of esRNAs, including microRNAs (miRNAs) and small interfering RNAs (siRNAs), have been identified both in metazoans and lower eukaryotic organisms [2–5]. Biogenesis of most of those esRNAs shares a minimal common machinery consisting in an RNase III endonuclease Dicer that processes double-stranded RNA (dsRNA) precursors into small RNA (sRNA) molecules, and an Argonaute endonuclease that binds sRNAs and uses them as a guide to identify and cleave complementary target mRNA. Additionally, some RNAi-competent organisms, including plants, nematodes and fungi, require RNA-dependent RNA polymerases to generate dsRNA from single-stranded RNA inducers or to amplify siRNA signals. Besides this canonical pathway, different non-canonical alternatives in which Dicer proteins do not participate have been described to be responsible for the biogenesis of specific esRNAs, not only the well-known Piwi-interacting RNAs (piRNAs) but also miRNAs and miRNA-like (milRNA) molecules [6–8]. In these cases, the catalytic activity of Argonaute family proteins and the trimming activity of specific exonucleases are required for the production of mature esRNAs. However, the majority of the non-canonical miRNA molecules are poorly conserved and low in abundance, which shed doubts on their functionality.

In filamentous fungi, different classes of regulatory esRNAs produced by canonical and non-canonical pathways have been described, although information on their functional roles is very scarce [3,9]. The basal fungus *Mucor circinelloides*, an emerging opportunistic human pathogen of the order mucorales [10], is a great model to investigate the functional roles of esRNAs, as shown by the finding of esRNAs derived from exons, named ex-siRNAs, that regulate the expression of the protein coding genes from which they derive [11]. Depending on the proteins of the RNAi machinery required for their biogenesis, these ex-siRNAs can be classified into four different classes, all of which are *dicer*-dependent, since they require one of the *M*. *circinelloides* Dicer-like (Dcl) proteins for their biogenesis. The main class of the *dicer*-dependent ex-siRNAs (class II) requires Dcl-2 [12] and RdRP-1 [13], an RNA-dependent RNA polymerase essential for activation of silencing with single stranded RNA molecules by producing antisense RNA transcripts. Only a small group of *dcl-2*-dependent ex-siRNAs (class I) does not require RdRP-1 for their biogenesis, but most of them require RdRP-2 [13], which is involved in the amplification process that produce secondary siRNAs. These two classes are specifically bound to Ago-1, one of the three *M*. *circinelloides* Argonaute proteins [14] and are accumulated at a reduced extent in the *ago-1* ^*-*^ mutants, suggesting that they require Ago-1 for their biogenesis/stability. Class III corresponds to ex-siRNAs that can be generated by either Dcl-1 or Dcl-2 and require both RdRP-1 and RdRP-2, and class IV is a tiny group of ex-siRNAs that depends on *dcl-1* [15] but not on *dcl-2* [11]. Classes III and IV are not found among Ago-1-bound ex-siRNAs, which suggests that either they do not act through a canonical pathway or they are bound to a different Ago protein [14].

The role of the *dicer*-dependent ex-siRNAs in the regulation of endogenous genes has been confirmed experimentally, since the reduction of specific ex-siRNAs in mutants affected in the RNAi machinery is associated with an increase in mRNA accumulation of the corresponding target protein coding genes [11,14]. In fact, RNA-seq analysis of *M*. *circinelloides* RNAi mutants identified hundreds of genes that showed differential mRNA expression compared to the wild type strain [16]. Detailed analysis of the differentially expressed genes allowed the identification of candidates that may be responsible for the phenotypes shown by mutants affected in the RNAi machinery, such as defects in vegetative growth, hyphal morphology and sporulation efficiency, or even differential response to nutritional stress [16,17]. Most of these phenotypes are related to developmental responses to endogenous and environmental signals, suggesting that the RNAi machinery modulates the expression of genes involved in these responses. This is supported by the ability of *M*. *circinelloides* to adapt to the environment through a new epigenetic mechanism based on an RNAi-mediated pathway [18], pointing out the relevance of the RNAi mechanism in the control of phenotypic plasticity.

Previous analyses of *M*. *circinelloides* esRNAs were exclusively focused on those produced through *dicer*-dependent pathways, since only esRNAs that showed a significant reduction in normalized reads in any of the *dcl* ^*-*^ mutants compared to wild type were considered [11,14]. On the other hand, comparisons of the phenotypes shown by the different RNAi mutants revealed that several phenotypes were shared by the *rdrp-1* ^*-*^ and *rdrp-2* ^*-*^ mutants but not the *dcl* ^*-*^ mutants [16]. We analyze here the complete esRNA content of the wild type, *dcl* ^*-*^ and *rdrp* ^*-*^ strains and identify a new *rdrp*-dependent *dicer*-independent esRNA class derived from exons. Analysis of these sRNAs shows that they are produced by a degradation pathway in which the RdRP-1 and/or RdRP-2 proteins mark specific transcripts to be degraded by a previously unknown RNase. Search of the *Mucor* genome for candidate RNases involved in this *rdrp*-dependent *dicer*-independent RNA degradation pathway and functional analysis of the corresponding genes identified a new protein, named R3B2. This RNase III-like protein presents a unique domain architecture and it is also implicated in the canonical *dicer*-dependent RNAi pathway, highlighting the crucial role of the R3B2 protein in the biogenesis and function of regulatory esRNAs. Phenotypic analysis of the *r3b2* ^*-*^ mutant and its comparison with other silencing mutants suggests that the *rdrp*-dependent *dicer*-independent degradation pathway regulates cellular responses to specific environmental signals.

## Results

### A new *rdrp*-dependent RNA degradation pathway in *M*. *circinelloides*

**Fig 1:**
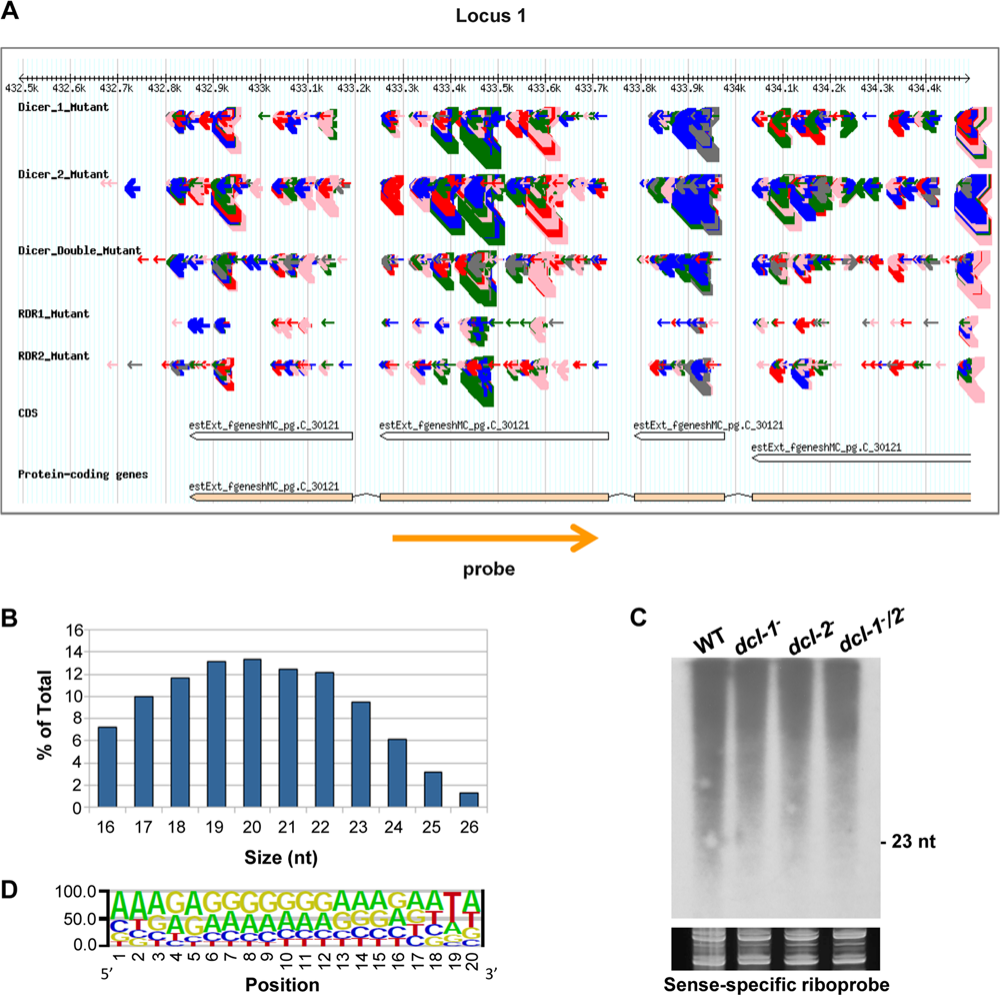
A new class of *dicer*-independent sRNAs in *M*. *circinelloides*. (A) Genome browser shot of an *rdrp*-dependent *dicer*-independent sRNA-producing locus showing sRNA accumulation in wt, *dcl-1* ^-^, *dcl-2* ^-^, *dcl-1* ^-^/*dcl-2* ^-^, *rdrp-1* ^-^ and *rdrp-2* ^-^ strains. Arrows represent sRNA sequence reads, which are exclusively sense to the mRNA. Thickness of the arrows indicates the abundance of read on a log scale. Color of arrows refers to the length of the sequence (pink <19 nt, red: 20–21 nt, green: 22–23 nt, blue: 24–25 nt and grey> 25nt). Orange arrow represents the position and orientation of the probe used for sRNA detection by Northern blots. The exon locus (locus 1) corresponds to the protein ID 90984, an alkaline phosphatase. (B) Size distribution of *rdrp*-dependent *dicer*-independent sRNAs in the wild type strain. The graph represents the percentage of raw reads corresponding to different length of sequenced sRNAs in the wild type strain. (C) Accumulation of sRNAs from the *rdrp*-dependent *dicer*-independent locus 1 in wild type and *dcl* ^-^ mutant strains. Low-molecular weight RNA (50 μg) was extracted from wild-type, *dcl-1^-^*, *dcl-2^-^* and *dcl-1^-^/dcl-2^-^* double mutant strains and probed with the sense-specific riboprobe shown in (A) (S2 Table). Ethidium bromide stained image of gel below the radiogram shows equal loading of lanes. Ten picomoles per lane of 23-mer to 25-mer DNA oligonucleotides in antisense and sense orientation were used as size markers and to control the hybridization specificity. No signal was detected with an antisense-specific riboprobe. (D) Sequence logo of the 20 nt size class of *rdrp*-dependent *dicer*-independent sRNAs.

We have previously demonstrated the existence of different classes of endogenous siRNAs (esRNAs) in *M*. *circinelloides* produced with the involvement of a Dicer activity [11]. The *ago-1*, *rdrp-1* and, at a minor extent, *rdrp-2* genes are also required, in different combinations, for the production of those esRNAs [11,14]. Many of these esRNAs derive from exons (ex-siRNAs) and regulate the expression of the protein coding genes from which they are produced [11,14]. Deep sequencing of short RNAs (18–25nt) in the *M*. *circinelloides* wild type, *dicer* ^*-*^ and *rdrp* ^*-*^ strains identified, besides the mentioned *dicer*-dependent esRNAs, new loci that produced esRNAs by a *dicer*-independent but *rdrp-1*- and/or *rdrp-2*-dependent mechanism. In these analyses, loci were defined by reads that mapped to the genome in close proximity (≤ 200 bp) to each other (as previously described in [11]). The *rdrp*-dependent *dicer*-independent sRNA loci were identified as those that showed at least a fourfold decrease in normalized sRNA reads in *rdrp-1* ^*-*^ or *rdrp-2* ^*-*^ mutants compared to wild type, with no significant change between wild-type and any of the *dicer* ^*-*^ mutants. We identified a total of 1523 *rdrp*-dependent loci, among which 611 were *dicer*-independent, and they were grouped based on the annotation of the locus as intergenic, transposon or exonic loci (Table 1 and S1 Fig). Whereas none or a small number of transposon and intergenic loci were *rdrp*-dependent *dicer*-independent, as many as 531 exonic loci corresponded to this category. These loci produced sRNAs that showed a very strong strand bias, almost all of them being exclusively sense to the mRNAs (Fig 1A, S1 Table and S2 Fig), but they did not show enrichment for a specific size (Fig 1B). This suggests these sRNAs are not produced by a canonical RNA silencing mechanism, since the majority of the known *M*. *circinelloides* ex-siRNAs are *dicer*-dependent and derive from exons producing a mixed sense and antisense ex-siRNAs mainly 23–24 nt long [11]. Most loci of the *rdrp*-dependent *dicer*-independent class required *rdrp-1* or both *rdrp-1* and *rdrp-2* genes for production of esRNAs (S1 Table and S1 Fig). Thus, despite the prominent role of the *rdrp-2* gene in transgene-induced silencing, *rdrp-1* seems to play a more relevant role in the production of endogenous sRNAs, both in a *dicer*-dependent [11] and in a *dicer*-independent pathway (Table 1).

**Table 1:** Number of loci down-regulated at sRNA level in *rdrp-1* ^-^ and *rdrp-2* ^-^ mutants^a^.

	Down-regulated in^b^			
Type of loci	*rdrp-1* ^-^	*rdrp-2* ^-^ ^c^	*rdrp-1* ^-^ and *rdrp-2* ^-^	Total
transposons	151 (0)	2 (0)	16 (0)	169 (0)
intergenic regions	424 (29)	12 (4)	71 (47)	507 (80)
exons	448 (223)	23 (20)	376 (288)	847 (531)
Total	1023 (252)	37 (24)	463 (335)	1523 (611)

^a^Total number of loci (with normalized abundance of more than 50 reads per million) showing a fourfold or higher reduction in the *rdrp* ^*-*^ mutant strains compared to the wild type.

^b^Loci that show the reduction only in the *rdrp-1* ^*-*^ or *rdrp-2* ^*-*^ mutants are considered separated from those with reduced levels of sRNAs in both *rdrp-1* ^*-*^ and *rdrp-2* ^*-*^ strains (see S1 Fig). Numbers in parentheses show the number of loci of each category that are *dicer*-independent. The *dicer*-dependent loci were considered in the esRNA classes already described [11].

^c^Most of the loci which are *rdrp-2*-dependent also showed a reduction in sRNA accumulation in the *rdrp-1* ^*-*^ mutant, although the values did not reach the threshold.

Accumulation of sRNAs from some of those *rdrp*-dependent *dicer*-independent loci was analyzed by Northern blot hybridization to validate the sequencing data. However, contrary to the *dicer*-dependent classes of esRNAs [11,14], we could not detect any sRNAs similar to those in the *dicer*-independent classes, neither with sense-specific or antisense-specific riboprobes (Figs 1C and S3). These sRNAs were either not detectable or the probes detected a smear between 15–2000-nt but not discrete bands with sizes between 20–25nt. Thus, we concluded that reads from these loci are most likely small degradation products of mRNAs. According to this, these *rdrp*-dependent *dicer*-independent sRNA molecules showed a random spread of size distribution (Fig 1B), different from the *dicer*-dependent classes that produced predominantly 23–24 nt sRNAs [11].

We analyzed the nucleotide distribution in each position of the *rdrp*-dependent *dicer*-independent sRNA degradation products and found a very strong bias for uracil in the penultimate position for all sizes of sRNAs (18–24 nt; Figs 1D and S4). This bias for uracil in the penultimate position, whereas it is under-represented in the rest of the sRNAs, suggests that the generation of this class of sRNAs is not random. In fact, if we extend the sequence upstream and downstream of the sRNAs, an over-representation of uracil can be detected 2 nt upstream of the sRNA for any size analyzed (S5 Fig), suggesting that an RNase may exist in *M*. *circinelloides* that preferentially cleaves mRNAs two nucleotides downstream of any uracil. This cleavage preference would produce fragments of various sizes resulting in the smears observed in the Northern blot analyses (Figs 1C and S3). However, when the distance between two uracils is 18–24 nucleotides, the cleavage products would be present in the sRNA library, as the libraries were generated from any 18–24-mer RNA molecules that can be ligated to adapters. Besides the preference for uracil in the penultimate position, sequence logos also showed a biased purine/pyrimidine distribution for all sizes of sRNA degradation products, which showed A/G enrichment throughout their whole sequence. This bias is not specific to *Mucor rdrp*-dependent sRNAs, since it is has been also found in canonical ex-siRNAs [11] and the small RNA datasets of seven different plant species [19]. It has been suggested that the distorted purine pyrimidine ratio in cellular sRNA populations implies that cells selectively accumulates purine rich strands and eliminates the pyrimidine rich strands, although the molecular mechanism for this active strand selection is not known [19].

In agreement with the non-canonical nature of the *rdrp*-dependent *dicer*-independent sRNAs, they do not show the strong bias for uracil at the 5’ end shown by the canonical ex-siRNAs bound by *M*. *circinelloides* Ago-1 [11,14]. In fact, only eleven out of 531 sRNAs of the *rdrp*-dependent *dicer*-independent class were detected among those specifically bound to *M*. *circinelloides* Ago-1 (S1 Table, [14]), suggesting that this sRNA class does not act through the canonical RNAi pathway. According to the nature of the *rdrp*-dependent *dicer*-independent RNA class, which was identified through sequencing small RNAs but corresponds to non-random degradation products of mRNAs that can have any size and are generated by a *rdrp*-dependent mechanism, we named those RNA molecules as ***r*** *drp*-dependent ***d***egraded RNA (rdRNA).

### The *rdrp*-dependent *dicer*-independent RNA degradation pathway regulates gene expression

**Fig 2:**
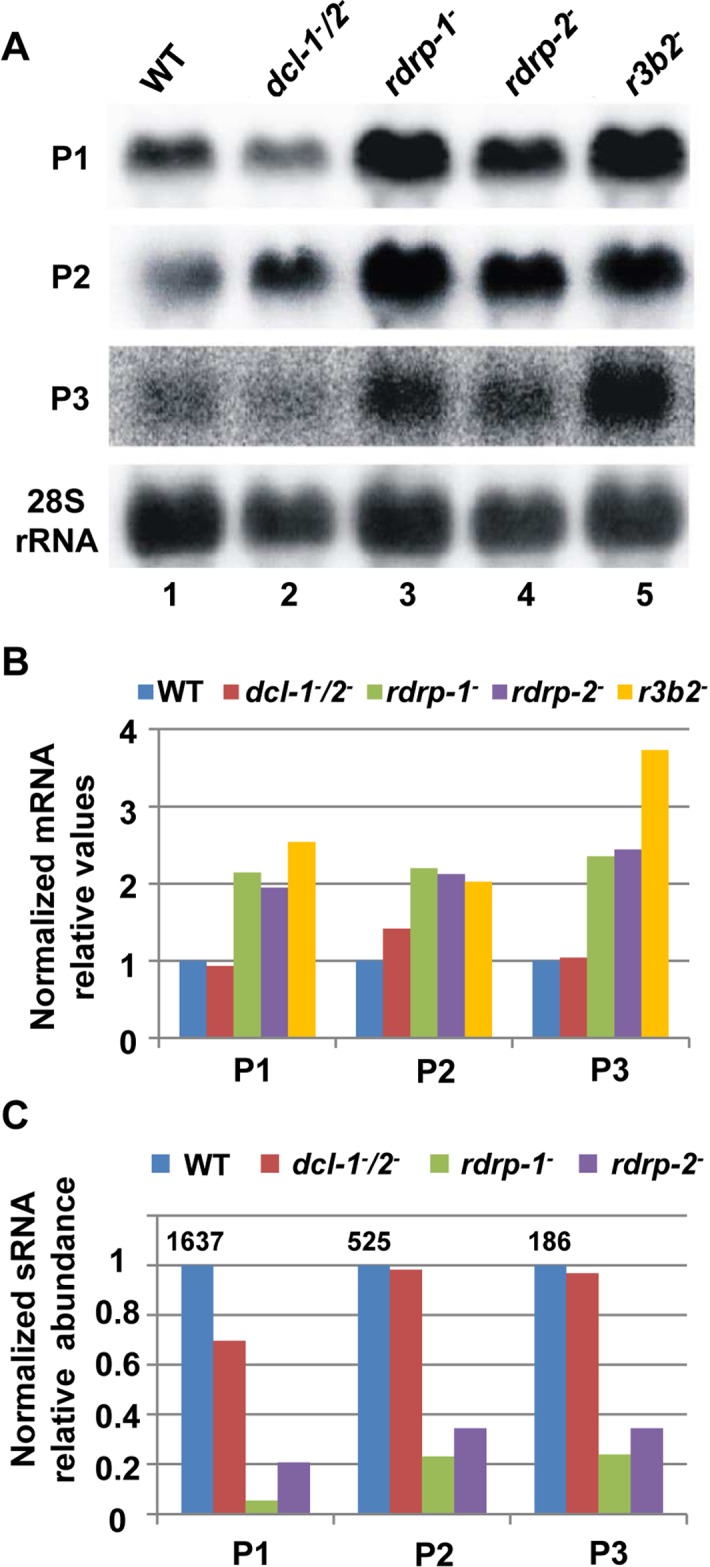
The *rdrp*-dependent *dicer*-independent RNA degradation pathway regulates gene expression. (A) Accumulation of mRNAs in wild type and silencing mutants. Northern blots of high molecular weight RNAs corresponding to rdRNA-producing exons (genes P1 to P3) were carried out using total RNA (50 μg) extracted from wild type, *dcl-* and *rdrp-* mutant strains (lanes 1–4) and a mutant affected in the ribonuclease gene *r3b2* (lane 5) grown for 24 hours in liquid MMC medium. Samples were separated in 1.2% denaturing agarose gel, transferred to membranes and hybridized with gene specific probes (S2 Table). The rdRNA-producing exon loci correspond to the following gene products: P1: ID 26072, pyruvate decarboxylase, P2: ID 92956, actin binding protein, P3: ID 92251, transmembrane protein similar to the B-cell receptor-associated protein Bap31 (S1 Table). The membranes were reprobed with a 28S rRNA probe as loading control. Images are representative of three independent experiments (B) Densitometric analysis of expression data shown in (A). Signal intensities were quantified and normalized to rRNA levels. All data were again normalized with respect to the expression value of the wild type strain (R7B) for each gene. (C) Normalized reads (abundance) in the *dcl-* and *rdrp-* mutant strains of rdRNAs corresponding to the exon loci P1 to P3 compared to wild type (R7B). All data were again normalized with respect to the abundance values of the wild type strain for each gene. Absolute normalized reads for each gene in the wild type strain are indicated. Locus coordinates: P1: scaffold_2/1367350-1369052; P2: scaffold_8/1301699-1302454; P3: scaffold_5/2811886-2812243.

Although the above results indicate that the *rdrp*-dependent *dicer*-independent rdRNAs are not "classical sRNAs" (i.e. they are not 20–24-mer RNA molecules generated by *dicer*), the sequencing data suggested that they are not non-specific degradation products, since accumulation of these rdRNAs was significantly reduced in the *rdrp* ^*-*^ mutants relative to the wild type and *dicer* ^*-*^ strains (S1 Table). This fact raised the possibility that this new pathway could also regulate the level of mRNAs. Therefore, we analyzed mRNA accumulation during the exponential growth from representative loci in the wild type, *rdrp* ^*-*^ and *dicer* ^*-*^ mutants by Northern blot analysis of RNA samples isolated from cultures grown 24 hours in liquid MMC medium. Three different rdRNA-producing exons (P1 to P3) were selected based on their different numbers of normalized sRNA reads in the wild type strain, thus representing the variability found in the sRNA transcriptomic analysis (Fig 2C). The accumulation of all tested mRNAs increased two fold, as an average, in the *rdrp* ^*-*^ mutant strains compared to the wild type and *dicer* ^*-*^ mutants when growing exponentially (Fig 2A and 2B, lanes 1–4). This demonstrated a clear trend of the reverse relationship between mRNAs and small RNAs accumulation of the selected reporter genes, the increase in mRNA levels being associated with the reduction in the normalized reads in the *rdrp* ^*-*^ mutants relative to the wild type and *dicer* ^*-*^ mutant strains (Fig 2C). A linear correlation between sRNA decrease and mRNA increase is not expected, given the different methods used for detection of mRNAs and sRNAs. sRNA read numbers are affected by the length of the mRNA, uracil content and distribution and the ligation bias [20], since only degradation fragments with a distance between two uracil of 18–24 nt would be able to be included in the sRNA libraries. However, if transcript accumulation of different genes is regulated by the same mechanism, it is expected that alteration of this mechanism by mutation provokes a similar effect in all those genes. The same results were obtained at stationary growth conditions, when cultures were grown for 48 hours in liquid MMC medium (S6A and S6B Fig, lanes 1–4). These results confirm the existence of a degradation pathway in *M*. *circinelloides* that regulates mRNA levels and requires RdRP-1 and/or RdRP-2 proteins but not Dcl-1 and Dcl-2.

**Fig 3:**
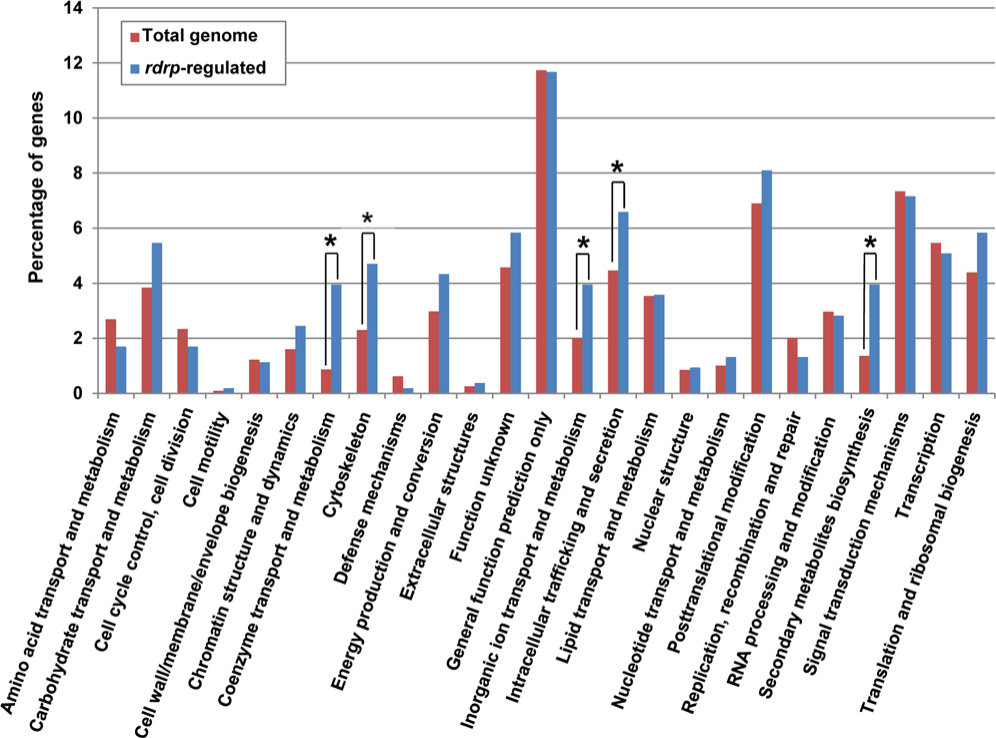
KOG classification of genes regulated by the *rdrp*-dependent *dicer*-independent rdRNAs. The distribution of genes regulated by the non-canonical *rdrp*-dependent *dicer*-independent RNA degradation pathway among the different Eukaryotic Orthologous Groups (KOG) classes (*rdrp*-regulated) is compared to the proportion of each class in the *M*. *circinelloides* whole genome (total genome). Asterisks indicate KOG classes showing significant enrichment in the *rdrp*-regulated genes relative to the total genome (Pearson's chi-squared test with Yates' continuity correction).

**Fig 4:**
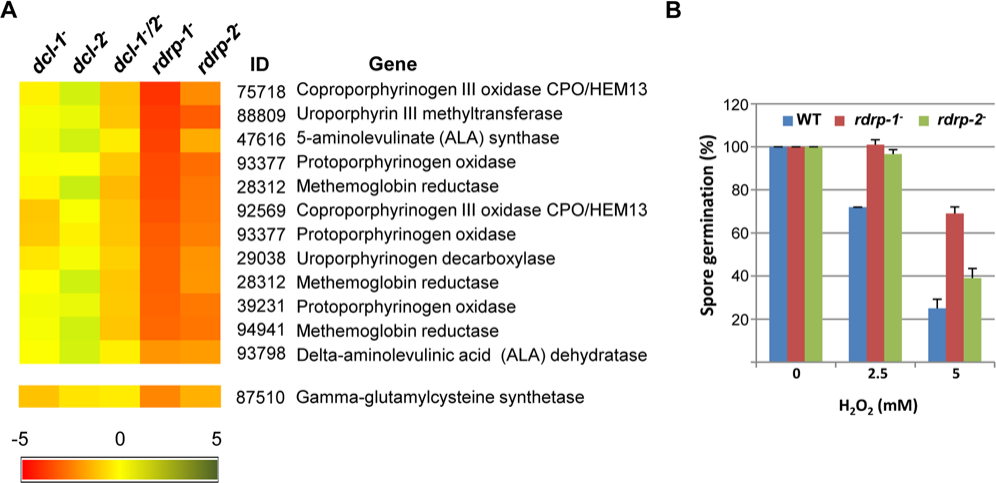
Differential regulation of genes involved in heme B biosynthesis or metabolism and oxidative stress response in *rdrp-* mutants. (A) Heat map showing the accumulation of rdRNAs from genes involved in heme B biosynthesis or metabolism. Accumulation of rdRNAs derived from the glutamate cysteine-ligase coding gene involved in the synthesis of the antioxidant glutathione is also shown. Each colored cell in the heat map represents the log_2_ fold change of rdRNAs in *dicer-* and *rdrp-* mutants compared to wild type strain. Expression levels above 0 represent up-accumulation, whereas those below 0 represent down-accumulation. Data were taken from S1 Table. (B) Oxidative stress response in *rdrp-1^-^* and *rdrp-2^-^* mutants. Spores of the wild type (R7B) and *rdrp-* mutant strains transformed to prototrophy with plasmid pLEU4 (*leuA* ^+^) were inoculated in YNB plates pH 3.2 containing different concentration of hydrogen peroxide (mM) and the percentage of germinated spores was calculated. The values are means and standard error of three independent experiments.

To identify the processes regulated by this new pathway, we performed an Eukaryotic Orthologous Group (KOG) enrichment analysis of the rdRNA-producing exons, according to the whole-genome annotation of *M*. *circinelloides*, whose version 2.0 is now available (http://genome.jgi-psf.org/Mucci2/Mucci2.home.html) (Figs 3 and 4, S1 Table). Although most of the KOG classes were similarly represented both among genes regulated by the non-canonical pathway and the total genome (Fig 3), we observed a significant enrichment in the regulated genes for those involved in coenzyme transport and metabolism, cytoskeleton, inorganic ion transport and metabolism, intracellular trafficking and secretion, and secondary metabolites biosynthesis, transport and catabolism. The most highly enriched class was the coenzyme transport and metabolism category, with 21 genes that account for 3.95% of the total number of regulated genes, compared to the 0.87% of these genes in the total genome (Fig 3). Twelve out of 21 genes of this class participate in heme B biosynthesis pathway or metabolism (Fig 4A). Besides haemoglobin and myoglobin, hemes are also found in a number of other biological relevant hemoproteins, such as catalase, which is an essential enzyme for protecting the cell from oxidative damage. Also the gene coding for gamma-glutamylcysteine synthetase (now glutamate cysteine-ligase, ID87510), which controls the first and rate-limiting step in the biosynthesis of the cellular antioxidant glutathione, is included in this class, accumulating a significantly lesser amount of rdRNAs in the *rdrp* ^*-*^ mutants relative to the wild type (Fig 4A). The differential expression of those genes in the *rdrp* ^*-*^ mutants could be responsible for the specific phenotypic alterations shown by those strains. In fact, the *rdrp-1* ^*-*^ and, at a lesser extent, *rdrp-2* ^*-*^ mutants are more resistant to oxidative stress than the wild type strain, as indicated by their ability to germinate in the presence of different concentrations of hydrogen peroxide (Fig 4B).

It is also worth noting the significant reduction in non-annotated genes among those regulated by the non-canonical pathway, with only 5.65% of non-annotated genes compared to 22.57% in the total genome (S1 Table). In fact, almost 60% of the rdRNA-producing loci correspond to conserved genes involved in metabolism and cellular processes and signaling (S7A Fig and S1 Table), which is significantly different to the functional annotation of genes regulated by the canonical *dicer*-dependent ex-siRNAs (S7B Fig, [11]). Thus, the canonical and non-canonical RNA pathways seem to regulate different groups of genes.

### Searching for candidate RNases in the *Mucor* genome and generation of knock-out mutants

The proposed pathway for the production of the rdRNAs involves the participation of an RNase that degrades target mRNAs. To identify the implicated RNase, we attempted to knock out four genes for putative RNAses: 80729, 136157, 110239, and 77996. Those proteins were identified by performing an *in silico* analysis of the *M*. *circinelloides* genome (v2.0) looking for annotated proteins containing RNase domains. Twenty-four proteins annotated under the endoribonuclease activity GO term (GO 0004521) were identified. A careful analysis of those proteins allowed us to select several candidates to be investigated for their participation in the non-canonical silencing pathway. Briefly, we selected putative RNases without precise information on their molecular role or those with functional annotation that could be related with the non-canonical pathway (S3 Table), discarding proteins with well-described ribonuclease activities, such as Dcl-1, Dcl-2, RNase P, RNase T2, RNase A, RNase H and others. Thus, the four putative RNases mentioned above were investigated. Because protein 80729 contains an RNaseIII domain (*r3*), and two dsRNA binding domain (*b2*), we named the corresponding gene *r3b2*. The selected genes and their adjacent sequences were amplified and knockout vectors were designed to disrupt each candidate gene by gene replacement (see description of plasmids and generation of knockout mutants in S1 Supporting Information). The knockout vectors contained the *pyrG* gene as selectable marker flanked by adjacent sequences of the different RNase genes to allow homologous recombination. Disruption fragments were used to transform the MU402 strain, which is auxotrophic for uracil and leucine, and transformants that correctly integrated the knockout fragment were identified by PCR analysis (S1 Supporting Information). After several vegetative cycles on selective media to increase the proportion of transformed nuclei, homokaryotic transformants were isolated and confirmed by Southern blot analysis (S1 Supporting Information). Two out of three homokaryotic transformants obtained with the *r3b2* disruption fragment were confirmed to contain a *pyrG* insertion of 3.4 kb replacing the *r3b2* gene (S8 Fig). One of them was named MU412 and was used as null mutant for this gene. Two out of four homokaryotic transformants obtained with the 136157 disruption fragment were confirmed as replacement mutants and were named MU450 and MU451 (S9 Fig). However, it was impossible to obtain homokaryotic knockout mutants for genes 110239 and 77996, as transformants containing the mutant alleles maintained wild type nuclei even after more than ten vegetative cycles on selective media (S10 Fig and S1 Supporting Information). This suggests that those genes may play essential roles for the viability of *M*. *circinelloides*.

### R3B2 is required for accumulation of *rdrp*-dependent *dicer*-independent rdRNAs

To investigate the role of the candidate RNases in the non-canonical RNA degradation pathway we first analyzed the accumulation of mRNA of representative loci regulated by this pathway (P1 to P3, see above) in the MU412 mutant (*r3b2* ^*-*^) and in the null mutants for gene 136157, MU450 and MU451. All tested mRNAs up-regulated in the *rdrp-1* ^*-*^ and/or *rdrp-2* ^*-*^ mutants compared to the wild-type strain and *dicer* ^*-*^ mutant were also up-regulated in the *r3b2* ^*-*^ mutant, in samples isolated during both exponential (Fig 2A, lane5) and stationary growth (S6A Fig, lane 5). In fact, the increase in mRNA accumulation of the target genes in the *r3b2* ^*-*^ mutant relative to the wild type strain was roughly two-fold, similarly to the *rdrp-1* ^*-*^ and *rdrp-2* ^*-*^ mutants (Figs 2B and S6B), suggesting that the *r3b2* gene encodes an RNase required for the degradation of specific mRNAs by the *rdrp*-dependent, *dicer*-independent non-canonical pathway. In contrast, mRNA accumulation of target genes in the MU450 and MU451 mutants, which are deficient in the putative RNase protein 136157, was similar to the wild type and *dicer* ^*-*^ mutant (S6C Fig), indicating that this RNase does not participate in the non-canonical RNA degradation pathway. Lack of homokaryotic null mutants for genes 110239 and 77996 precluded the analysis on their participation in the *rdrp*-dependent, *dicer*-independent pathway.

The presumed role of the RNase III R3B2 in the generation of rdRNAs by the *rdrp*-dependent, *dicer*-independent non-canonical pathway was confirmed by deep sequencing of the sRNA content (18–25 nt) in the *r3b2* ^*-*^ mutant and its comparison with the wild type strain (accession number SRR1576768). Almost 1,560 exonic loci were identified that showed a significant reduction in normalized sRNA reads in the *r3b2* ^*-*^ mutant relative to the wild type (S5 Table). Those loci were selected as those showing at least a fourfold decrease in normalized reads in the *r3b2* ^*-*^ mutant compared to wild type, and a normalized abundance count of more than 50 in the wild type. All but one of the 531 rdRNA-producing loci were found among those significantly down-regulated in the *r3b2* ^*-*^ mutant, as shown in Tables 2 and S6. In fact, the log_2_ fold change values in the *r3b2* ^*-*^ mutant relative to the wild type strain were even more significant than those of the *rdrp* ^*-*^ strains for most of the *rdrp*-dependent *dicer*-independent exonic loci, pointing to the relevant role of R3B2 in the biogenesis of rdRNAs. The lower rdRNA levels in the *r3b2* ^*-*^ mutant can be also seen in genes involved in heme biosynthesis, which show at least a 38-fold reduction in rdRNA accumulation relative to the wild type strain (S11 Fig). These results demonstrate the participation of the R3B2 protein in the biogenesis of the *rdrp*-dependent *dicer*-independent rdRNAs, strongly suggesting that it is indeed the RNase involved in the degradation process of specific mRNAs by the non-canonical silencing pathway.

**Table 2:** Accumulation of different classes of exonic sRNAs in the *r3b2* ^-^ mutant^a^.

sRNA class		No. of exons	% of down-regulated in *r3b2* ^*-*^	Average log_2_ fold change *r3b2* ^*-*^ vs WT^a^
***dicer*-independent (rdRNAs)**	*rdrp-1* and *rdrp-2*-dependent	288	100	**-6.24**
	*rdrp-1*-dependent	223	100	**-5.70**
	*rdrp-2*-dependent	20	95 (19 out of 20)	**-3.73**
***dicer*-dependent** ^b^ **(ex-siRNAs)**	Class I	9	33.3 (3 out of 9)	-0.07
	Class II	222	66.2 (147 out of 222)	**-2.57**
	Class III	88	97.7 (86 out of 88)	**-6.92**
	Class IV	5	80 (4 out of 5)	**-5.24**

^a^Average value of the log_2_ fold change of the different classes of exonic sRNAs in the *r3b2* ^*-*^ *M*. *circinelloides* mutant compared to wild type. Log_2_ fold changes in S6 (*dicer*-independent) and S7 (*dicer*-dependent) Tables were used to calculate the averages. Numbers in bold indicate a higher than four-fold down-regulation in the *r3b2* ^*-*^ mutant relative to wild type.

^b^ *dicer*-dependent ex-siRNAs are classified as previously described [11,14].

### R3B2 also participates in the canonical *dicer*-dependent RNA silencing pathway

Different types of *dicer*-dependent ex-siRNAs were previously identified and classified based on the components of the silencing machinery required for their biogenesis [11]. Most of those classes were also found among the exonic sRNAs significantly down-regulated in the *r3b2* ^*-*^ mutant (Tables 2 and S7). The majority of ex-siRNAs of the *dicer*-dependent classes II, III and IV showed at least a four-fold reduction in the *r3b2* ^*-*^ mutant relative to the wild type, whereas only three out of nine loci of class I were significantly reduced in the mutant strain. These results indicate that R3B2, besides its role in the non-canonical pathway, also participates in the production of the majority of canonical *dicer*-dependent ex-siRNAs, although its contribution varies among the different ex-siRNA classes. Particularly interesting is the large decrease in ex-siRNAs of class III in the *r3b2* ^*-*^ mutant. Class III ex-siRNAs can be produced both by Dcl-1 and Dcl-2, since their reduction is only seen in the double *dcl-1* ^*-*^/*dcl-2* ^*-*^ mutant, and its biogenesis requires the participation of both RdRP-1 and RdRP-2 proteins [11]. The structural characteristic of this ex-siRNA class and its lack of binding to Ago-1 indicated that class III ex-siRNAs are not *bona fide* ex-siRNAs and it had been proposed that they could be produced by degradation of specific mRNAs by unknown RNases [11,14]. The structural and functional similarities between class III of *dicer*-dependent ex-siRNAs and the *rdrp*-dependent *dicer*-independent rdRNAs and the large decrease of both classes in the *r3b2* ^*-*^ mutant might suggest that they can be produced by the same RNase, the R3B2 protein. In fact, comparing the reduction of the class III ex-siRNA levels in the double *dcl-1* ^*-*^/*dcl-2* ^*-*^ mutant (average log_2_ fold change from wild type -3.21 [11]) with their reduction in the *r3b2* ^*-*^ mutant (-6.92; Table 2) suggests that R3B2 plays the more prominent role in the production of this class of ex-siRNAs (see Discussion).

To confirm the participation of the *r3b2* gene in the *dicer*-dependent canonical pathway, we analyzed the capacity of the *r3b2* ^*-*^ mutant to activate the silencing mechanism by exogenous sequences. For that, we transformed the *r3b2* ^*-*^ mutant and the wild type strain with two different self-replicative silencing vectors containing sequences of the *carB* gene (phytoene dehydrogenase) expressed from the strong promoter of the *gpd1* gene (glycerol-3-phosphate dehydrogenase) as silencing reporter, since *carB* function is required for the production of colored carotenoids. Plasmid pMAT1279 contains a sense *carB* transgene (s-transgene) [13], whereas plasmid pMAT1253 expresses a *carB* hairpin RNA (hpRNA) [12]. Both plasmids were able to efficiently activate silencing of the endogenous *carB* gene in the wild type strain, giving rise to a high proportion of transformants that remained albino in the light, because of the absence of the *carB* function (Table 3). However, the frequency of albino transformants was severely reduced in the *r3b2* ^*-*^ mutant, in which only a few colonies with albino patches were obtained. These results indicated that *r3b2* is required for efficient transgene-induced silencing regardless the nature of the silencing trigger, since sense and inverted repeat transgenes showed a similar reduction in the efficiency of silencing compared to the wild type strain. As expected, null mutants for the candidate RNase 136157 (MU450 and MU451 strain), which does not participate in the non-canonical silencing pathway (S6C Fig), showed silencing frequencies similar to the wild type strain (Table 3), indicating that this putative RNase does not play any role in the canonical silencing pathway either.

**Table 3:** Gene silencing by sense and inverted repeat transgenes in candidate RNase mutants^a^.

		No of transformants^d^			
Plasmid^b^	Strain	Albino	Bright yellow	Total	Silencing frequency (%)
pMAT1279 (s-transgene)	wild-type	90	23	113	79.6
	MU412 (*r3b2* ^*-*^)	5	103	108	**4.6**
	MU450	17	5	22	77.2
	MU451	65	12	77	84.4
pMAT1253 (hpRNA)	wild-type	130	21	151	86.0
	MU412 (*r3b2* ^*-*^)	14	206	220	**6.4**
	MU450	50	2	52	96.1
	MU451	51	9	60	85
pMAT771 (hpRNA/*r3b2* ^*wt*^)	wild-type	57	204	261	21.8
	MU412 (*r3b2* ^*-*^)	44	111	155	28.4
pMAT772 (hpRNA/r3b2*)	wild-type	76	211	287	26.5
	MU412 (*r3b2* ^*-*^)	10	215	225	**4.4**
pLEU4 (control)^c^	wild-type	0	312	312	0
	MU412 (*r3b2* ^*-*^)	0	79	79	0
	MU450	0	17	17	0
	MU451	0	11	11	0

^a^Strain MU412 is the null mutant for the *r3b2* gene. Mutants MU450 and MU451 are affected in the candidate RNase 136157.

^b^hpRNA, construct expressing hpRNA; s-transgene, construct expressing the sense transgene.

^c^Control plasmid without a silencing construct

^d^The colours of the *M*. *circinelloides* transformants were observed after 48 h under illumination with white light. Colonies with patches of albino and wild type (bright yellow) phenotype were considered as albino, since most of the silenced primary transformants showed patches of different phenotypes due to the presence of several nuclei in the *M*. *circinelloides* protoplasts, but they turned uniformly albino after a cycle of vegetative growth in selective medium.

### The RNase-like domain is essential for accurate R3B2 function in RNA silencing

**Fig 5:**
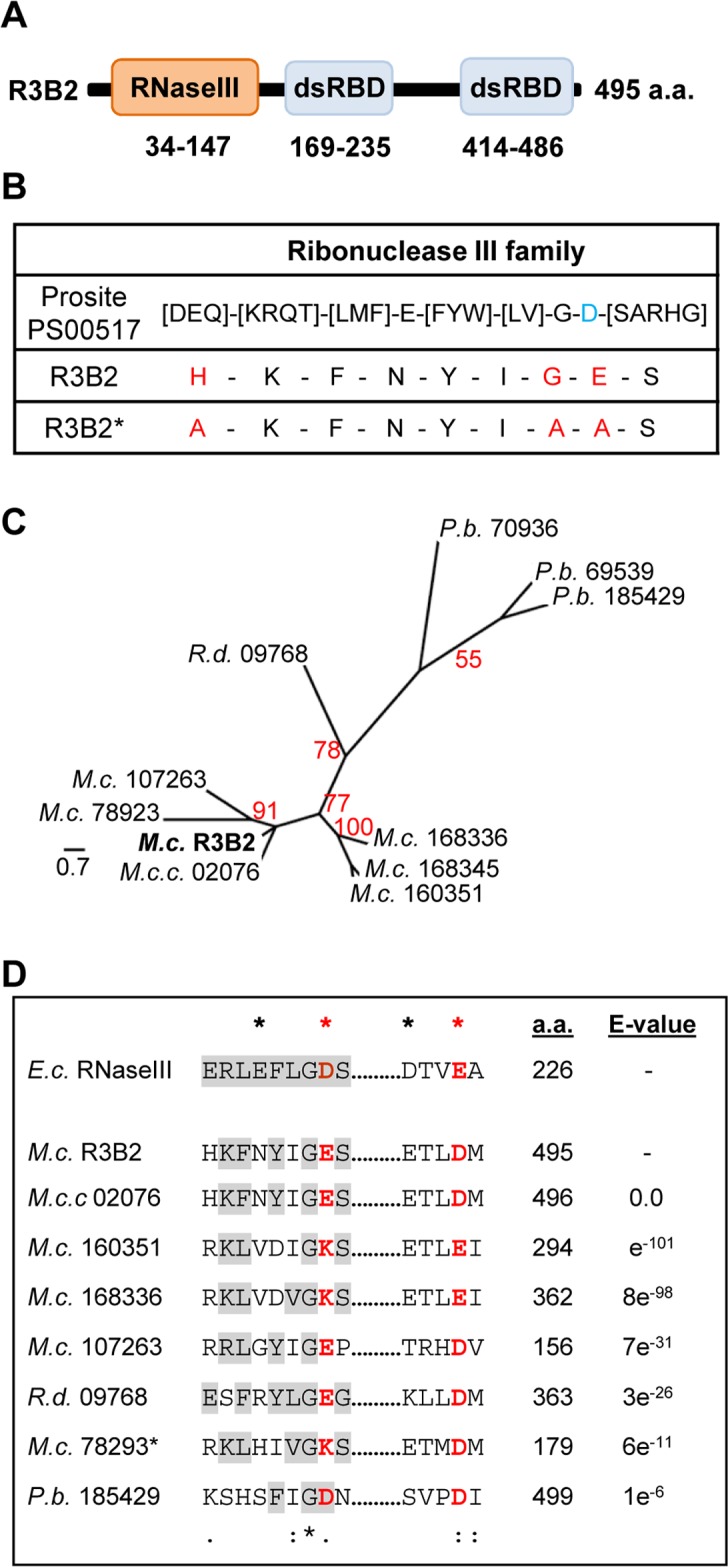
Domain structure and conserved residues of the *M*. *circinelloides* RNase III R3B2. (A) Domain organization of the R3B2 protein. Domains are shown by boxes with the starting and stopping amino acid of each domain indicated. RNase III: RNase III catalytic domain-like; dsRBD: dsRNA binding domain. (B) Signature motif of the Ribonuclease III family (Prosite PS00517). The conserved aspartic acid residue that has been demonstrated to be required for catalytic activity *in vitro* [21] is shown in blue. The corresponding sequences in the wild type R3B2 and mutant R3B2* proteins are shown below the signature, with the residues that have been changed in the mutant protein marked in red. (C) Phylogenetic relationship of *M*. *circinelloides* R3B2 protein (M.c.R3B2) and other mucoralean proteins. Phylogenetic tree was constructed using PhyML v3.0 aLRT method (maximum likelihood) [47] from sequence alignment created using MUSCLE 3.7 with default setting [48], using the Phylogeny software (http://www.phylogeny.fr) [49]. Branch lengths are proportional to the number of substitutions per site (bars). The numbers at the nodes are bootstrap values (%) for 100 replications. FungiDB accession number of the proteins were: *M*. *circinelloides* QYA_80729 (*M*.*c*. R3B2), QYA_160351 (*M*.*c*. 160351), QYA_168336 (*M*.*c*. 168336), QYA_168345 (*M*.*c*. 168345), QYA_107263 (*M*.*c*. 107263) and QYA_78293 (*M*.*c*. 78293), *Rhizopus delemar* RO3G_09768 (*R*.*d*. 09768) and *P*. *blakesleeanus* PHYBL_185429 (*P*.*b*. 185429), PHYBL_70936 (*P*.*b*. 70936) and PHYBL_69539 (*P*.*b*. 69539). GeneBank accession number of the *M*. *circinelloides f*. *circinelloides* protein was HMPREF1544_02076 (*M*.*c*.*c*. 02076). (D) Amino acid residues of the R3B2 protein family in conserved regions of the RNase III domain. Signature motif in the RNase III domain and the catalytic residues of the *Escherichia coli* RNase III are shown on the top. The strictly conserved acidic residues that coordinate metal binding are marked by asterisks, the red ones being demonstrated to be essential for catalysis in *E*. *coli* [21]. *E*. *coli* sequences correspond to residues 38–46 and 114–118 (SWISS-PROT P0A7Y0). The amino acid residues of the corresponding regions in the R3B2 protein family are shown below. Residues matching the signature motif are highlighted in grey. The number of amino acid residues of each protein and the E-value in BLAST comparison using R3B2 as query are shown.

The R3B2 protein (ID 80729) is annotated in the *M*. *circinelloides* genome (v2.0) as containing an amino-terminal RNase III catalytic domain-like of the SCOP (Structural Classification of Proteins) superfamily SSF 69065, and two C-terminal dsRNA-binding domains (Fig 5A). Comparison of the RNase III catalytic domain-like of R3B2 with the Ribonuclease III family signature (Prosite PS00517) identified several substitutions in conserved amino acids (Fig 5B). In fact, the invariant glutamic acid in the signature is changed to asparagine in R3B2 and the aspartic acid residue that is essential for catalysis *in vitro* [21] is substituted by glutamic acid.

To confirm that the R3B2 function in RNA silencing relies on its RNase III domain-like, we performed directed mutagenesis to change several residues of the domain and analyzed the ability of the mutant allele to complement the lack of R3B2 function in the *r3b2* ^*-*^ null mutant. The R3B2 residues H49, G55 and E56, which correspond to the highly conserved E38, G44 and D45 residues of the *E*. *coli* RNase III [21], were simultaneously changed to alanine (Fig 5B) (S1 Supporting Information). This *r3b2* mutant allele (r3b2*) was cloned in a *M*. *circinelloides* vector that expresses a *carB* hairpin RNA (hpRNA) under the control of the *gpd1* promoter, giving rise to plasmid pMAT772 (S1 Supporting Information). As a control, plasmid pMAT771, which contains a wild type *r3b2* allele and the hairpin *carB* transgene, was also constructed. Those plasmids were used to transform the wild type and null *r3b2* ^*-*^ mutant strains and the ability of the transformants to silence the expression of the endogenous *carB* gene was analyzed. Transformation of the *r3b2* ^*-*^ mutant with the control plasmid pMAT771 should simultaneously complement the null *r3b2* ^*-*^ mutation and induce silencing of the *carB* gene. In fact, the silencing frequency in the *r3b2* ^*-*^ mutant when transformed with this complementing plasmid was similar to the wild type strain (Table 3), demonstrating that the *r3b2* wild type allele is perfectly able to complement lack of R3B2 function in the *r3b2* ^*-*^ mutant strain. The reduction observed in the efficiency of pMAT771 to induce silencing relative to other silencing vectors is probably due to the large size of this plasmid. This could result in a low plasmid copy number in the transformants, which has been demonstrated to negatively affect silencing efficiency [22]. A similar silencing frequency was obtained when the silencing vector containing the r3b2* mutant allele (plasmid pMAT772) was used to induce silencing in the wild type strain. However, this plasmid was barely able to activate silencing in the *r3b2* ^*-*^ mutant, indicating that substitutions of conserved residues in the RNase III domain-like of R3B2 greatly abolish the activity of this protein in the canonical transgene-induced RNA silencing pathway.

**Fig 6:**
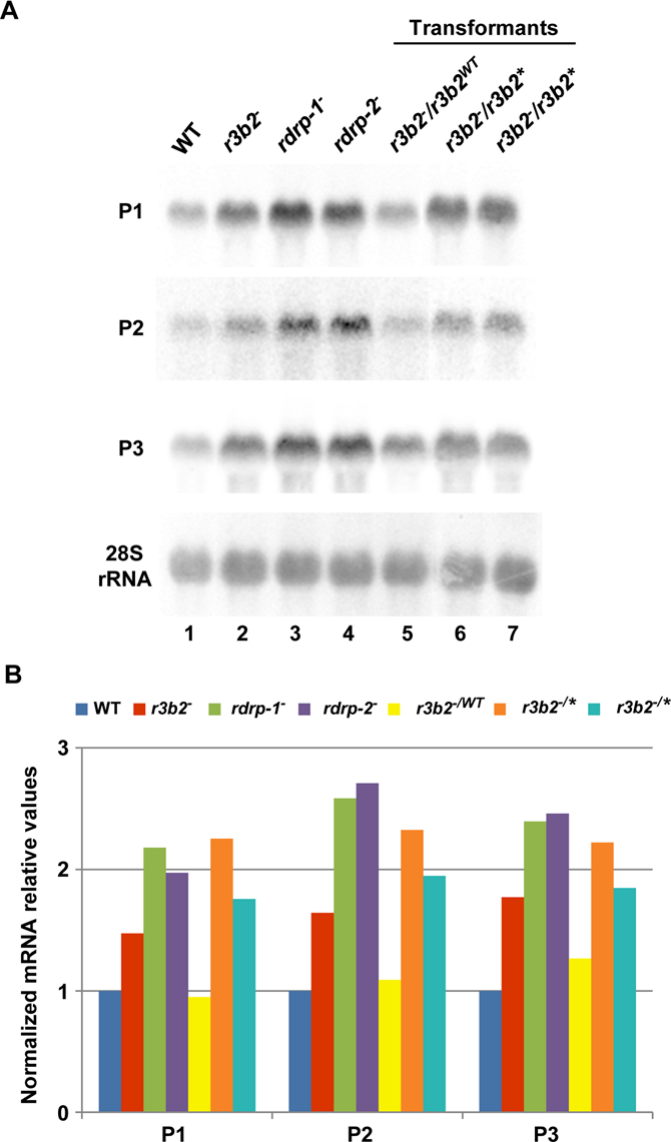
The RNase III domain-like of R3B2 is essential for R3B2 function. (A) Accumulation of mRNAs in *r3b2-* transformants harboring *r3b2* wild type or mutant alleles integrated at the *carRP* locus. Northern blots of high molecular weight RNAs corresponding to rdRNA-producing exons (genes P1 to P3) were carried out using total RNA (50 μg) extracted from wild type (R7B), *r3b2-* and *rdrp-* mutant strains (lanes 1–4) and transformants of the *r3b2-* mutants containing the wild type *r3b2* allele (*r3b2WT*, lane 5) or the *r3b2* allele carrying mutations in conserved residues of the RNaseIII-like domain (r3b2*, lanes 6 and 7) grown for 24 hours in liquid MMC medium. Samples were separated in 1.2% denaturing agarose gel, transferred to membranes and hybridized with gene specific probes (S2 Table). Genes P1 to P3 correspond to those indicated in Fig 2. Wild type and mutant *r3b2-* transformants correspond to transformants 6, 3 and 5 in S12 Fig, respectively. Membranes were reprobed with a 28S rRNA probe as loading control. Images are representative of two independent experiments. (B) Densitometric analysis of expression data shown in (A). Signal intensities were quantified and normalized to rRNA levels. All data were again normalized with respect to the expression value of the wild type strain (R7B) for each gene.

To confirm the requirement of an intact RNase III-like domain for the R3B2 function in the non-canonical RNA degradation pathway we constructed stable strains containing the wild type and r3b2* mutant alleles integrated at the *carRP* locus. Integration at the *carRP* locus can be easily detected due to the color change provoked by the disruption of the *carRP* gene, which encode a bifunctional enzyme with phytoene synthase and lycopene cyclase activities [23]. For the integrative complementation analysis, disruption fragments containing the wild type and mutant r3b2* alleles flanked by sequences of the *carRP* locus were constructed (plasmids pMAT787 and pMAT788, S1 Supporting Information) (S12A Fig). Those fragments were used to transform the null *r3b2* ^*-*^ mutant strains and transformants that remain albino in the light were selected, since integration at the *carRP* locus provokes the disruption of the *carRP* gene and avoids accumulation of colored carotenoids. Several homokaryotic transformants harboring the wild type *r3b2* or mutant r3b2* alleles integrated at the *carRP* locus were obtained (S12B Fig), and they were used to analyze mRNA accumulation of genes regulated by the *rdrp*-dependent *dicer*-independent pathway. The *r3b2* wild type allele integrated at the *carRP* locus efficiently complements the effect of the *r3b2* ^*-*^ mutation on mRNA accumulation of target genes, since all tested genes up-regulated in the *r3b2* ^*-*^, *rdrp-1* ^*-*^ and *rdrp-2* ^*-*^ mutants recovered their wild type expression levels in the complemented strain (Fig 6A and 6B, lane 5). However, the *r3b2* ^*-*^ transformants harboring the r3b2* mutant allele integrated at the *carRP* locus showed an increased mRNA accumulation of the target genes similar to the recipient strain (Fig 6A and 6B, lanes 6 and 7), demonstrating that the r3b2* mutant allele was unable to complement the *r3b2* ^*-*^ mutation. Together, those results indicate that the RNase-like domain of R3B2 is required for the correct function of this protein both in the canonical *dicer*-dependent RNAi pathway and in the *rdrp*-dependent *dicer*-independent RNA degradation pathway.

### The *r3b2* ^*-*^ mutant exhibits differential response to environmental signals

**Fig 7:**
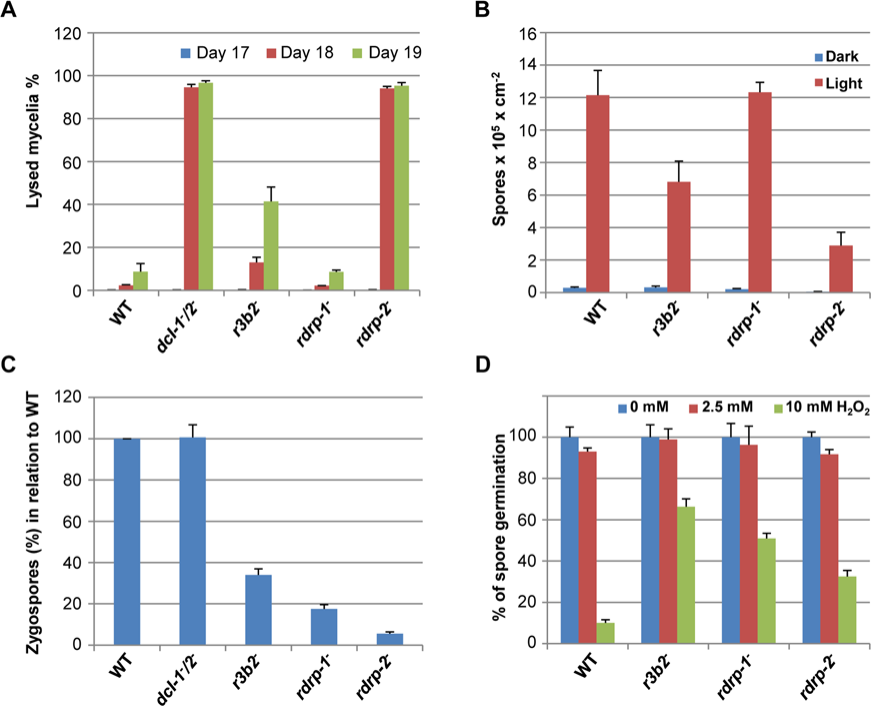
Phenotypes of *M*. *circinelloides r3b2^-^* mutant. (A) Autolysis in the *r3b2-* mutant. Percentages of lysed mycelia relative to the total mycelial area of the wild type R7B strain and *r3b2-* mutant are shown. Strains were grown in rich YPD medium under dark conditions from spores inoculated in the center of Petri dishes until mycelia covered the complete plate area (2–3 days). After different times of incubation (17–19 days), the lysed area of each mycelium relative to the total mycelial area was measured. The values are mean and standard errors of four independent measurements. Data from silencing mutants showing accelerated autolysis (*dcl-1^-^/dcl-2^-^* and *rdrp-2^-^* mutants) and from non-affected mutants (*rdrp-1^-^*) [14] are shown for comparison. (B) Asexual spore production in the *r3b2-* mutant. Production of asexual spores in mycelia of the wild type strain R7B and the *r3b2-* mutant grown under white light (red bars) and dark (blue bars) conditions for 3 days in solid MMC medium pH 3.2 is shown. The values are means and standard errors of 10 independent measurements. Data from affected (*rdrp-2^-^*) and non-affected (*rdrp-1^-^*) mutants are shown for comparison. (C) Zygospore production by the *r3b2-* mutant. Relative zygospore production in genetic crosses between the wild type and *r3b2-* mutant (-) strains and the wild-type NRRL3631 (+) is shown. Data are the averages and standard errors of the zygospores quantified in twelve histological sections (0.003 cm^2^) of the sexual interaction area for each cross after normalized against the zygospores produced in crosses between wild-type strains. Data from crosses between non-affected (*dcl-1^-^/dcl-2^-^*) and affected (*rdrp-1^-^* and *rdrp-2^-^*) mutants and the wild type NRRL3631 (+) strain are included for comparison [16]. (D) Oxidative stress response in the *r3b2-* mutant. Spores of the wild type strain (R7B) and *r3b2-* and *rdrp-* mutants were inoculated in YNB plates pH 3.2 containing different concentration of hydrogen peroxide (0, 2.5 and 10 mM) and the percentage of germinated spores was calculated. The values are means and standard error of three independent experiments.

Confirming the participation of R3B2 in both, the canonical and non-canonical RNA pathways, the null *r3b2* ^*-*^ mutant presented phenotypes associated to alterations in cellular processes controlled by those pathways (Fig 7). The *dcl-2* ^*-*^, *ago-1* ^*-*^ and *rdrp-2* ^*-*^ mutants, which participate in the canonical *dicer*-dependent ex-siRNA pathway, are affected in cellular processes connected with nutrient sensing of the cells, such as production of vegetative spores and autolysis induced by nutrient starvation [12,14,16]. Those processes are also affected in the *r3b2* ^*-*^ mutant, which presents intermediate phenotypes both, for the autolysis of aged mycelia provoked by nutritional stress, which initiates at earlier state than the wild type strain (Fig 7A) and for the production of vegetative spores, which is significantly reduced relative to the wild type (Fig 7B). Even more relevant is the corroboration of the R3B2 participation in the *rdrp*-dependent *dicer*-independent RNA degradation pathway. Besides their better response to oxidative stress (Fig 4), the *rdrp* ^*-*^ mutants showed defects in sexual interaction and production of zygospores [16]. These phenotypes had been observed only in *rdrp-1* ^*-*^ and *rdrp-2* ^*-*^ mutants but not in *dcl* ^*-*^ or *ago-1* ^*-*^ mutants, suggesting that a non-canonical *dicer*-independent RNA pathway had to be involved. The *r3b2* ^*-*^ mutant is also affected in the production of zygospores (Fig 7C) and showed an increased resistance to oxidative stress relative to the wild type (Fig 7D), confirming its participation in the *rdrp*-dependent *dicer*-independent RNA degradation pathway and suggesting a role for this pathway in the response to specific environmental signals.

### An RNase family specific to mucorales

The domain architecture of R3B2 is unusual, since prokaryotic and fungal class 1 RNase IIIs contain only one dsRNA binding domain, besides the RNase III catalytic motif, whereas classes 2 and 3 of eukaryotic RNase III are larger proteins with several structural domains, as occurs in Drosha and Dicer [24]. In fact, no proteins with the same domain architecture as R3B2 could be identified in the Conserved Domain Architecture Retrieval Tool (CDART) [25]. To investigate the presence of proteins similar to R3B2 in the fungal kingdom, the fungal and oomycete genomics resource FungiDB (http://fungidb.org/fungidb/) [26] was used. Sixty four organisms from 14 fungal classes are included in this data base, which allows searching for genes using different criteria. Searching for proteins similar to R3B2 identified nine proteins of this database with an expected value lower than one, all of them belonging to the order mucorales (S13 Fig). No other proteins were identified when using less stringent conditions, indicating that, within the fungal kingdom, the R3B2 protein family seems to be specific of the order mucorales. Most of the proteins identified contain RNase III-like and/or dsRNA binding domains, although the majority of them are smaller than R3B2. The phylogenetic relationship among the R3B2 protein family shows several *M*. *circinelloides* paralogous proteins highly similar to R3B2 (Fig 5C). It is not known if those proteins are expressed and if their structure has been correctly annotated. However, it could be possible that one or several of them might play accessory roles in RNA silencing pathways, since they contain similar residues at the catalytic sites of their RNase III-like domains as R3B2 (Fig 5D). In fact, most of the proteins of the R3B2 family contain acidic residues at the catalytic positions, although three out of four *M*. *circinelloides* R3B2 paralogs have a positively charged lysine residue in one of these positions, raising doubts about their functionality. Moreover, none of the R3B2 paralogs have been annotated as containing RNase III domains in the *Mucor* genome, suggesting that their differences with the consensus sequence for this domain is too high to allow their detection as putative RNase III. We also investigated the presence of proteins similar to R3B2 among sequences included at the National Center for Biotechnology Information Server (NCBI). No proteins, except those present in the publicly available *M*. *circinelloides f*. *circinelloides*1006PhL (ID HMPREF1544_02076) and *Rhizopus delemar* RA 99–880 (ID RO3G_09768) genomes were identified (S13 Fig). Strikingly, the RNase III-like domain of R3B2 showed a limited similarity (best e-value 0.15) with the RNase III domain of different bacteria of the order Burkholderiales, although the domain of these bacterial proteins contains all the conserved residues of the RNase III signature. These data could suggest a horizontal transfer event between *Burkholderia* and an ancestor of the order mucorales and the generation of a fusion protein, with subsequent duplications and diversifications in different mucoralean lineages.

## Discussion

### A novel pathway to regulate mRNA accumulation

Several RdRP proteins have been involved in the production of endogenous siRNAs in plants and nematodes [4,27]. In those organisms, RdRPs show functional diversification in distinct endogenous silencing pathways as they are linked to the action of specific Dicer enzymes and/or Argonaute proteins. Also in *M*. *circinelloides* the two RdRP proteins described are functionally different. The RdRP-1 protein is involved in activation of silencing by sense transgenes and produces antisense RNAs corresponding to transgene transcripts [13]. It is also required for the production of the largest class of *dicer*-dependent ex-siRNAs [11]. RdRP-2 is involved in the amplification process that produces secondary siRNAs [13] and it has a role in the production of several classes of *dicer*-dependent ex-siRNAs [11]. We have shown here that, besides playing an essential role in this endogenous silencing pathway, the RdRP enzymes are also involved in a novel mechanism that control degradation of specific mRNAs. By deep sequencing, more than 500 loci corresponding to exons were observed to produce short RNAs in an *rdrp*-dependent but *dicer*-independent manner. However, no discrete RNA species could be detected by northern blot, suggesting that they may be degradation products of mRNAs. Sequence analysis of these short RNA molecules and their flanking genomic regions indicated that this degradation was not random and suggested the existence of a *M*. *circinelloides* RNase that preferentially cleaves mRNAs two nucleotides downstream of any uracil. Different RNase-based mechanisms have been involved in the control of mRNA stability but an RdRP enzyme was not demonstrated to participate in any of those mechanisms [28]. Our results indicate that RdRP-1 and/or RdRP-2 proteins have a functional role in the degradation of specific mRNAs, since the levels of these mRNAs were significantly increased in *rdrp* ^*-*^ mutants. Thus, reduction of the mRNA degradation rate in the *rdrp* ^*-*^ mutants would be associated with a low accumulation of degradation products (rdRNAs), leading to the identification of the corresponding loci as *rdrp*-dependent. Confirming the non-canonical nature of this *rdrp*-dependent *dicer*-independent rdRNAs, only a minority of them were found associated with Ago-1, the *M*. *circinelloides* Argonaute protein involved in exogenous and endogenous RNAi canonical pathways [14]. Although two other *ago* genes have been identified in *Mucor*, their genomic sequences and expression patterns do not suggest a role for their protein products in the degradation pathway described in this study [14].

We have analyzed the biological functions of genes regulated by this novel degradation pathway (S1 Table). Although the large number of affected genes makes it difficult to precisely understand the processes regulated by this degradation pathway, it can be emphasized that many of those genes code for metabolic enzymes or proteins involved in regular cellular functions, such as mRNA processing, translation or signaling. Particularly interesting is the regulation of genes involved in heme biosynthesis or metabolism (Fig 4). Heme B, the most abundant heme, is synthetized by eight enzymatic steps, some of which occur in the cytoplasm and some in the mitochondrion [29] (S14A Fig). Five out of eight proteins involved in heme biosynthesis are regulated by the *rdrp*-dependent *dicer*-independent degradation pathway (S14B Fig), suggesting a role for this pathway in the regulation of heme-containing protein(s). Curiously, a *M*. *circinelloides* protein (ID 95051) highly similar to the ferrochetalase enzyme, which is required to bind iron to protoporphyrin IX, is regulated by *dicer*-dependent ex-siRNAs of class III (S7 Table), which share some characteristics with the rdRNAs (see below). In addition to that, the uroporphyrinogen III methyltransferase, which controls the first of the three steps leading to the formation of siroheme from uroporphyrinogen III, is also regulated by this pathway (S14 Fig). Siroheme is a heme-like prosthetic group for sulfite and nitrite reductases that is required for methionine and cysteine synthesis [29]. Finally, two proteins highly similar to methemoglobin reductases, which are involved in heme metabolism by reducing the iron in the heme group from the ferric state (methemoglobin) to the ferrous state of the normal hemoglobin, are also regulated by the *rdrp*-dependent *dicer*-independent degradation pathway (S14 Fig). Impairment of the degradation pathway in the *rdrp* ^*-*^ mutants would result in an increased accumulation of the mRNAs corresponding to the mentioned genes and thus, an up-regulation of heme biosynthesis and, consequently, an increase of intracellular heme levels. In fungi, hemes are found in a number of biological relevant proteins, i.e. peroxidases, cytochrome, flavohemoglobins and others [29]. Many of these proteins are involved in the response to different environmental stresses, such as low oxygen conditions [30]. Interestingly, one of the most relevant fungal hemoproteins is catalase, which is essential for protecting the cell from oxidative damage [31].

The *M*. *circinelloides* glutamate cysteine ligase-like protein 87510 is also regulated by the *rdrp*-dependent *dicer*-independent degradation pathway (Fig 4). This enzyme, also named gamma-glutamylcysteine synthetase, catalyzes the first and rate-limiting step in the production of cellular antioxidant glutathione, which plays key roles in the response to several stress situations in fungi, including oxidative stress [32]. Several other genes regulated by the *rdrp*-dependent *dicer*-independent pathway also encode antioxidant proteins, such as thioredoxin (ID 87683), glutaredoxin (ID 37397) and peroxiredoxin (ID 25842 and ID 51186). Up-regulation of these genes in the *rdrp* ^*-*^ and *r3b2* ^*-*^ mutants, together with the increase in heme biosynthesis, could explain the better response to oxidative stress shown by these strains relative to the wild type, manifested by their increased ability to germinate in presence of hydrogen peroxide (Figs 4B and 7D). Detailed analysis of each of the above genes would be required to assess their specific roles in the responses of *M*. *circinelloides* to oxidative stress and other environmental signals. On the other hand, the high number of genes regulated by the *rdrp*-dependent *dicer*-independent pathway containing domains involved in transcriptional regulation or signal transduction, makes difficult to ascertain the gene(s) responsible(s) for the sexual behavior of the *rdrp-1* ^*-*^, *rdrp-2* ^*-*^ and *r3b2* ^*-*^ mutants. However, it is worth noting that one of the genes regulated by this pathway codes for a protein (ID 43858) highly similar to the mating factor M secretion protein Mam1 of *Schizosaccharomyces pombe* (1.3E^-125^), which is responsible for the secretion of the mating pheromone [33]. It is tempting to speculate that modulation of *M*. *circinelloides* protein expression in mutants affected in the *rdrp*-dependent *dicer*-independent pathway could be responsible, at least in part, of the defects shown by those mutants in their sexual behavior.

### The role of R3B2 in canonical and non-canonical RNA silencing pathways

**Fig 8:**
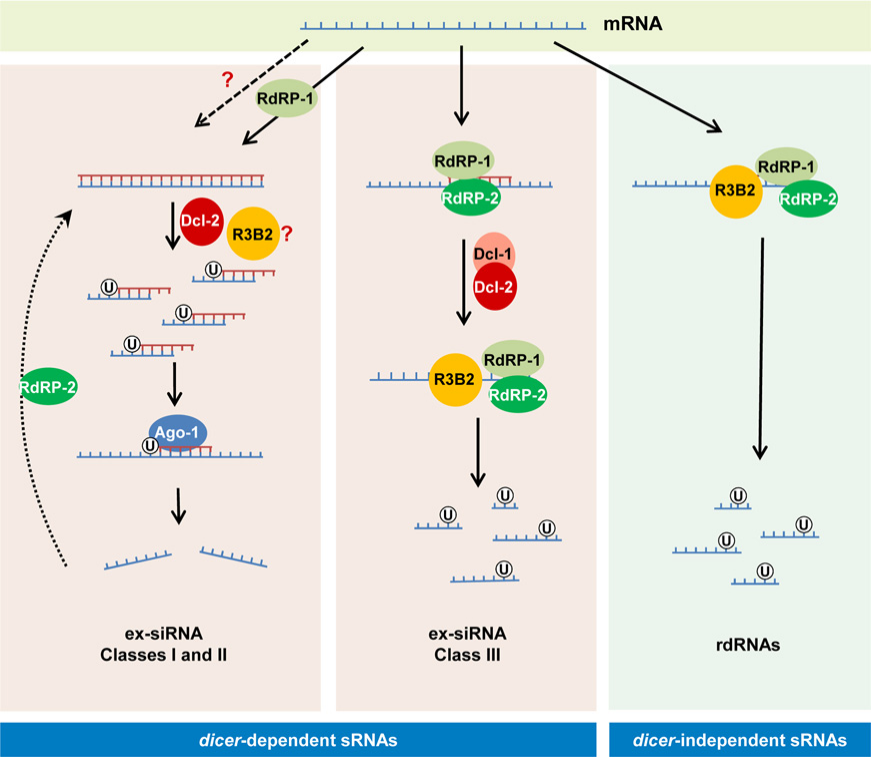
Models for production of the different classes of endogenous sRNAs in *M*. *circinelloides*. The *dicer*-independent sRNAs identified in this work (right) derive from specific transcripts that are targeted for degradation by RdRP-1 and/or RdRP-2 binding. The RdRP proteins bound to these transcripts may be able to make a short complementary strand that signals them for degradation by the RNase III-like protein R3B2. This protein preferentially cleaves mRNAs two nucleotides downstream of any uracil, giving rise to different size fragments with a uracil in the penultimate position (rdRNAs). The *dicer*-dependent ex-siRNAs can be classified into four classes depending on the component of the silencing machinery required for their biogenesis. Classes I and II (left) correspond to Ago-1-bound ex-siRNAs that are processed by Dcl-2 from dsRNAs generated by the action of RdRP-1 (class II, solid arrows) or RdRP-2 (Class I, broken arrows). R3B2 plays a role in the production of some of these ex-siRNAs, although its specific role is not yet known. Classes I and II are canonical ex-siRNAs with a defined size of 23–24 nt and a preference for uracil at the 5’ position. The structural characteristics of class III ex-siRNAs (middle) are similar to those of rdRNAs, suggesting that these ex-siRNAs are also degradation products generated by R3B2. The involvement of Dcl-1 or Dcl-2 in the biogenesis of class III ex-siRNAs suggests that the discrete dsRNA stretches produced by RdRP proteins must be processed by either Dcl-1 or Dcl-2 and after the initial cleavage the single stranded portions of mRNAs would be degraded by R3B2. Class IV ex-siRNAs, which require Dcl-1 and both RdRP-1 and RdRP-2 proteins for their biogenesis, are not indicated since they represent a small number of ex-siRNAs derived from only five exons.

We have identified the RNase III-like protein R3B2 as the RNase involved in the *rdrp*-dependent *dicer*-independent RNA degradation pathway. More than 1,500 exonic loci were identified that produced sRNAs in a R3B2-dependent manner (S5 Table). These loci included all but one *rdrp*-dependent *dicer*-independent loci, revealing the participation of this RNase in the degradation pathway (S6 Table). Surprisingly, also a significant number of *dicer*-dependent ex-siRNA loci, mainly those belonging to the class III ex-siRNAs, were found to be dependent on R3B2 for their biogenesis (S7 Table). Class III ex-siRNAs share several structural and functional features with the *rdrp*-dependent *dicer*-independent rdRNAs [11,14]. They have the same polarity as mRNA and a random spread of size distribution, as well as a very strong preference for uracil in the penultimate position, and they do not specifically bind to Ago-1, as occurs with the rdRNAs. This allowed us to propose that class III ex-siRNAs are not produced by a canonical RNAi pathway [11]. The difference with the *rdrp*-dependent *dicer*-independent rdRNAs relies in the participation of Dcl-1 or Dcl-2 in the biogenesis of class III ex-siRNAs. Thus, it can be suggested that the activity of RdRP-1 and/or RdRP-2 on target transcripts, presumably aberrant transcripts lacking normal processing signals such as a 5′ cap or a polyA tail, generates discrete dsRNA stretches that could be directly recognized by the RNase III-like R3B2, targeting those transcripts for degradation (rdRNAs) or could be firstly processed by either Dcl-1 or Dcl-2 and after the initial cleavage the single stranded portions of mRNAs would be degraded by R3B2 (class III ex-siRNAs) (Fig 8).

R3B2 contains a catalytic RNase III domain and two dsRBDs, which differ from typical class 1 RNase IIIs in terms of the number of dsRBDs. Only a single protein from *Arabidopsis thaliana*, the RNase III-like protein 2 (AtRTL2, At3g20420) display this unusual domain organization, although its catalytic domain contains a canonical RNase III signature motif [34]. Class 1 RNase IIIs are normally involved in the processing of ribosomal RNA precursors and some mRNAs. However, AtRTL2 has no effect on rRNA maturation *in vivo*, which suggests that it may function differently to RNase IIIs of class 1 [35]. In fact, this protein cleaves dsRNAs *in vitro* giving rise to cleavage products of longer size than other class 1 RNases, and it is involved in the production of small RNAs derived from transgenes *in vivo*. This raised the possibility that AtRTL2 may interact with other *A*. *thaliana* Dicer enzymes to positively affect the Dicer activity in siRNA generation [35]. Although the sequence similarity between AtRTL2 and *M*. *circinelloides* R3B2 is low, their similar and unique domain organization supports that R3B2 also has functions distinct from those of other class 1 RNase IIIs *in vivo*. Results shown here indicate that the RNase III domain-like of R3B2 is required for efficient R3B2 function in RNA silencing, although it lacks a canonical RNase III signature motif. The biochemical requirements for RNA cleavage by R3B2 are unknown, but the presence of two dsRBD, which might be utilized for protein–protein interactions [36], suggests that it may interact with other members of the RNAi machinery (e.g. RdRP or Dicer proteins) to degrade target transcripts or positively affect siRNA production.

### A genetic link between mRNA degradation and post-transcriptional RNA silencing

Most genes regulated by the *rdrp*-dependent *dicer*-independent pathway seem to be highly expressed, as denoted by the high number of sequences derived from those genes that are included in the EST repertoire sequenced from *M*. *circinelloides* (http://genome.jgi-psf.org/Mucci2/Mucci2.home.html). For instance, all but one of genes involved in heme biosynthesis and metabolism shown in Fig 4 are present in the *Mucor* EST collection, whereas the global percentage of genes with ESTs in the *Mucor* genome is only 33%. It is tempting to speculate that this RdRP-dependent degradation process is a control mechanism for genes with high levels of expression, since elevated transcription increases the production of aberrant RNAs [37,38]. All eukaryotic cells, including fungi, contain general and specialized mRNA decay pathways that target aberrant transcripts for degradation. Besides these quality control systems, the correct RNA turnover of mRNAs, carried out by defined degradation mechanisms, can play an important role in setting the basal level of mRNA expression and how that level is modulated by environmental stimuli [39]. Although numerous components of the RNA degradation mechanisms have already been identified, no RdRP enzyme has been demonstrated to be involved in any of those mechanisms. However, several evidences suggest that proteins involved in proper mRNA turnover or RNA quality-control systems compete with the RNAi machinery for aberrant transcripts [40–42]. In all the reported cases, the efficiency of transgene-induced gene silencing increased in mutants affected in the mRNA degradation pathways, suggesting that degradation of aberrant transcripts limits their entry into the RNAi pathway and providing insights into the interplay between mRNA degradation and post-transcriptional gene silencing. Here, we have shown a genetic link, the *rdrp* genes, between these two processes. The RdRP proteins bound to aberrant transcripts may be able to either make a short complementary strand that signals the RNase R3B2 for preferential degradation or synthesize long dsRNA molecules that trigger the RNAi mechanism. How the RdRP enzymes discriminate what RNAs are directed to the canonical silencing pathway or to the degradation pathway is not known yet. However, it is worth noting that the results obtained in this work agree with the enhanced activation of the RNAi-induced epimutation pathway observed in the *rdrp-1* ^*-*^ mutants [18]. It has been shown that spontaneous resistance to an antifungal drug via an epigenetic RNAi-mediated pathway that silences the drug target gene is highly increased in the *rdrp-1* ^*-*^ mutants, suggesting that in these mutants the RNA degradation pathway has been abolished and mRNAs are primarily directed to the canonical RNAi silencing pathway. The involvement of RdRPs in RNA degradation could represent the first step in the evolution of the RNAi mechanism. RNAi is a complex process and it is unlikely that the entire process developed at once. The RdRP could be the first player that somehow marked mRNAs for degradation. Later on, Dicer may have appeared and cleaved the RdRP products. Finally, Argonaute proteins evolved to acquire the siRNAs produced by Dicer and use them for further RNA degradation. It is tempting to speculate that in *M*. *circinelloides*, and probably other members of the mucoralean basal lineage of the fungal kingdom, all of these mechanisms are still simultaneously operating.

## Materials and Methods

### Strains, growth and transformation conditions

The leucine auxotroph R7B, derived from the (-) mating type *M*. *circinelloides f*. *lusitanicus* CBS 277.49 (syn. Mucor racemosus ATCC 1216b), was used as the wild type strain. Strain MU402 [15] is a uracil and leucine auxotroph derived from R7B used as recipient strain to knock out the candidate RNase genes. Strains MU411 (*dcl-1* ^*-*^ */dcl-2* ^*-*^) [12], MU413 (*ago-1* ^-^) [14], MU419 (*rdrp-1* ^*-*^) and MU420 (*rdrp-2* ^*-*^) [13] were all derived from MU402. The *M*. *circinelloides f*. *lusitanicus* strain of the (+) mating type NRRL3631 was used in sexual interaction analysis. Cultures were grown at 26°C in minimal YNB medium, complete YPG medium or in MMC medium as described previously [15]. Media were supplemented with uridine (200 μg/ml) or leucine (20 μg/ml) when required. The pH was adjusted to 4.5 and 3.2 for mycelial and colonial growth, respectively. Transformation was carried out as described previously [43]. For increasing the proportion of transformed nuclei, transformants were grown in selective medium for several vegetative cycles, since primary transformants are heterokaryons due to the presence of several nuclei in the protoplasts. Illumination conditions were as previously described [44]. Competent cells of *E*. *coli* DH5α strain were used for cloning experiments.

### Plasmids

A complete description of plasmids used in this work for cloning and functional analysis of the candidate RNase genes can be found in S1 Supporting Information.

### Nucleic acid manipulation and analysis

Genomic DNA from *M*. *circinelloides* mycelia was extracted as previously described [15]. Recombinant DNA manipulations were performed as reported [45]. To identify transformants that correctly integrate the knockout vectors designed to disrupt the candidate RNase genes, a rapid protocol for isolating DNA to be used in PCR amplifications was utilized [15]. Total RNA was isolated using Trizol reagent following the supplier’s recommendation (Invitrogen). Southern blot and Northern blot hybridizations were carried out under stringent conditions [15]. DNA probes were labeled with [α-^32^P]dCTP using Ready-To-Go Labeling Beads (GE Healthcare Life Science). For Northern blot experiments, P1, P2 and P3 probes were directly amplified from genomic DNA using specific primers (S2 Table). For Southern blot hybridizations, specific probes that discriminate between the wild type and disrupted alleles of each candidate RNase gene were obtained as follows. The *r3b2* probe (S8 Fig, probe *a*) corresponds to a 720 bp *Sac*I fragment isolated from plasmid pMAT1294, which contains the *r3b2* gene and adjacent sequences (S1 Supporting Information). The 136157 probes *b* and *c* (S9 Fig), correspond to a 1.1 kb *Hinc*II and 0.6 kb *Nco*I/*Sac*I fragments isolated from plasmid pMAT767, respectively. The 110239 specific probe (S10 Fig, probe *d*) corresponds to a 0.8 kb fragment of its downstream region amplified by the primer pair F6/R5 (S4 Table). The 77996 probe (S10 Fig, probe e) corresponds to a 1.0 kb fragment of its downstream region amplified by the primer pair F9-pyrG/R8 (S4 Table). The *carRP* probe (S12 Fig, probe f) used to identify homologous integration of *r3b2* alleles into the *carRP* locus was amplified with primers carRP-F1 and carRP-R2 (S4 Table). Signal intensities were estimated from autoradiograms using a Shimadzu CS-9000 densitometer and the ImageJ application, an open source image analysis program (rsbweb.nih.gov/ij/). EcoTaq Plus (Ecogen, Spain) or PfuUltra Hotstart DNA Polymerase (Stratagene) were used in PCR experiments.

### Cloning of candidate RNase genes and generation of knock-out mutants

A PCR-based strategy was used for cloning the selected genes encoding proteins with an RNase domain and for the generation of knock-out vectors to disrupt each gene. A precise description of the constructs and the procedures used can be found in S1 Supporting Information.

### Phenotypic analysis

Vegetative sporulation and lysis measurements were carried out as previously described [14]. Autolysis of aged mycelia was estimated by image analysis of the plates using ImageJ (rsbweb.nih.gov/ij/). Quantification of sexual mating and zygospores formation was carried out as described [16]. Briefly, spores of the (-) and (+) mating types were co-inoculated in the middle of agar YPD plates, approximately 2 cm apart, and incubated at room temperature under dark conditions during 20 days. The formation of zygospores in the contact zone gives rise to a dark line that was sliced in portions of 1 cm^2^ and fixed in 10% formaldehyde during 10 hours. After fixation, samples were frozen and sliced using a cryotome to produce sections of 30 μm. Zygospores from twelve sections were counted by optical microscopy (bright field 10X) for each interaction.

### Small RNA analysis

For endogenous sRNA analysis, small RNA samples were extracted from mycelia grown 48 h on YPG plates using the miRVana kit (Ambion), following the instructions of the supplier. cDNA libraries of small RNAs were generated and sequenced as described previously [11]. For isolation of sRNAs bound to the Ago-1 protein, small RNA samples were extracted from Ago-1-containing fractions and used to construct the cDNA library as previously described [11]. Equivalent fractions from the *ago-1* ^*-*^ mutant were used for isolation of sRNAs as a negative control. Sequencing of the Ago-1 bound cDNA libraries were carried out as described [14]. For detection of endogenous sRNAs in Northern blot experiments, membranes were hybridized as described [22] with sense and antisense-specific riboprobes prepared by *in vitro* transcription (MAXIscript transcription kit; Ambion) of linearized plasmids containing specific sequences for each locus (S2 Table).

### Sequence analysis

Computational sequence analysis was carried out using European Bioinformatics Institute Server software (EMBL Outstation, Hinxton, U.K.), the National Center for Biotechnology Information Server (NCBI, Bethesda, MD, USA) and the Methodes et Algorithmes pour la Bio-informatique LIRMM (MABL) server (Montpellier, France).

For the analysis of endogenous sRNAs, raw reads were processed and normalized as previously described [11,14]. sRNAs were mapped to annotated exons, transposons and intergenic regions of the *M*. *circinelloides* genome (http://genome.jgi-psf.org/Mucci1/Mucci1.home.html) (v 1.0) using PatMaN [46]. sRNA loci were said to be down-regulated in a given sample if the normalized locus abundance showed at least a fourfold decrease in comparison to the wild type sample (log_2_ fold change ≤ -2). This arbitrary fourfold difference was used as a cut-off to increase the stringency of the analysis. sRNAs were said to be bound to Ago-1 if the normalized abundance in the Ago-1 fractions purified from the wild type strain showed at least a fourfold increase relative to the *ago-1* ^*-*^ sample (log_2_ fold change ≥ 2). To increase the stringency of the analysis and avoid lowly expressed regions, any loci with a normalized abundance count of less than 50 in the wild type were excluded from the analysis.

### Nucleotide sequence accession number

The accession numbers of the sRNAs cloned in the wild type and *rdrp-1* ^*-*^ and *rdrp-2* ^*-*^ mutants are GSM469403, GSM469406 and GSM469407, respectively; all accessions are under GEO accession GSE18958. The raw reads of *M*. *circinelloides* Ago-1-bound small RNAs in wild type and *ago-1* ^*-*^ mutant are deposited in the Sequence Read Archive (SRA) database under the accession number SRR835448. The raw reads of *M*. *circinelloides* small RNAs in the *r3b2* ^*-*^ mutant have been deposited in the SRA database under the accession number SRR1576768.

## Supporting Information

S1 Fig Venn diagrams representing the number of loci that require *dicer*, *rdrp-1* and/or *rdrp-2* to generate the endogenous sRNAs. All loci dependent on any of the *dicer* genes (*dcl-1-*, *dcl-2-* or *dcl-1*/*dcl-2-*dependent) are included in the same category (*dcl* ^*-*^).
(PDF)

S2 Fig Genome browser shots of selected loci. Several *rdrp*-dependent *dicer*-independent sRNA-producing loci were randomly selected to show sRNA accumulation in wt, *dcl-1* ^-^, *dcl-2* ^-^, *dcl-1* ^-^/*dcl-2* ^-^, *rdrp-1* ^-^ and *rdrp-2* ^-^ strains. Arrows represent sRNA sequence reads (thickness and color references as in Fig 1). Orange arrows represent the position and orientation of probes used for sRNA detection by Northern blots. The exon loci correspond to the following proteins: locus 2: ID 46819, inorganic phosphate transporter; locus 3: ID 11610, unknown protein; locus 4: ID 94060, phosphatase involved in carbohydrate transport and metabolism; locus 5: ID 30368, heat shock protein; locus 6: ID 77714, Dipeptidyl aminopeptidase; locus 7: ID 29487, succinate dehydrogenase, flavoprotein subunit.
(PDF)

S3 Fig Accumulation of sRNAs from *rdrp*-dependent *dicer*-independent exonic loci in wild type and *dcl* ^-^ mutant strains. Low-molecular weight RNA (50 μg) was extracted from wild-type, *dcl-1* ^*-*^, *dcl-2* ^*-*^ and *dcl-1* ^*-*^ */dcl-2* ^*-*^ mutant strains and probed with sense and antisense-specific riboprobes specific to each locus (S2 Table; see S2 Fig). Ethidium bromide stained images of gels below the radiograms show equal loading of lanes. Ten picomoles per lane of 23-mer to 25-mer DNA oligonucleotides in antisense and sense orientation were used as size markers and to control the hybridization specificity. In all cases, the RNA probes hybridized to these controls. Results obtained for three representative loci are shown (see S2 Fig). The antisense-specific riboprobes did not give any signal in any of the loci analyzed.
(PDF)

S4 Fig Sequence logo of *rdrp*-dependent *dicer*-independent sRNAs. The frequency of each of the four bases was calculated in *rdrp*-dependent *dicer*-independent exonic sRNAs. The result is shown for separate sizes of 18–24 nts. The numbers on the x-axis refer to the position in the sRNAs and the y-axis shows the percentage distribution. The top to bottom order of bases in each position is determined by their frequency (highest on top). The figure shows that uracil (T in the cDNA sequences) is highly enriched at the penultimate position and under-represented in the rest of the sRNA sequence.
(PDF)

S5 Fig Extended sequence logo of *rdrp*-dependent *dicer*-independent sRNAs. The frequency of each of the four bases was calculated 5-bp upstream and 5-bp downstream of the sRNA. The result is shown for separate sizes of 20–24 nts. Red lines mark the ends of the sRNA sequence. The numbers on the x-axis refer to the position in the genomic sequence and the y-axis shows the percentage distribution. The top to bottom order of bases in each position is determined by their frequency (highest on top). The figure shows that uracil (T in the cDNA sequences) is highly over-represented in the position -2 relative to the sRNA 5’-end.
(PDF)

S6 Fig Accumulation of mRNAs in wild type and silencing mutants. (A) Northern blots of high molecular weight RNAs corresponding to rdRNA-producing exons (genes P1 to P3) were carried out using total RNA (50 μg) extracted from wild type, *dcl* ^*-*^ and *rdrp* ^*-*^ mutant strains (lanes 1–4) and a mutant affected in the ribonuclease gene *r3b2* (lane 5) grown for 48 hours in MMC medium. Samples were separated in 1.2% denaturing agarose gel, transferred to membranes and hybridized with gene specific probes (S2 Table). Genes P1 to P3 correspond to those indicated in Fig 2. The membranes were reprobed with a 28S rRNA probe to check loading. Images are representative of two independent experiments (B) Densitometric analysis of expression data shown in (A). Signal intensities were quantified and normalized to rRNA levels. All data were again normalized with respect to the expression value of the wild type strain (R7B) for each gene. (C) Accumulation of mRNA from the rdRNA-producing exons (P1 to P3) in mutants affected in the 136157 RNase. Total RNA (50 μg) extracted from the MU450 and MU451 mutants, affected in the 136157 RNase gene, as well as the wild type strain and *dcl* ^*-*^ and *rdrp* ^*-*^ mutants grown for 24 hours in liquid MMC medium was hybridized with gene specific probes (S2 Table). The membrane was reprobed with a 28S rRNA probe as loading control. Images are representative of two independent experiments. (D) Densitometric analysis of expression data shown in (C). Signal intensities were quantified and normalized as in (B).
(PDF)

S7 Fig Biological process categories of genes regulated by the *rdrp*-dependent *dicer*-independent RNA degradation pathway. (A) The proportion of genes regulated by the *rdrp*-dependent *dicer*-independent non-canonical pathway within the different KOG biological process categories is shown. Data were taken from S1 Table. (B) Similar analysis of genes regulated by canonical *dicer*-dependent ex-siRNAs is shown for comparison. Data were taken from [11].
(PDF)

S8 Fig Disruption of the *r3b2* gene. (A) Schematic representation of the wild-type *r3b2* locus (middle) and after homologous recombination with the disruption fragment (below). Yellow and blue boxes represent genomic *r3b2* locus and adjacent sequences, respectively; red boxes, *pyrG* selectable marker; dashed lines, sequences not included in the disruption fragment. The *Xba*I and *Xho*I sites used to release the disruption fragment from the knockout vector pMAT1298 are indicated (see S1 Supporting Information). The positions of the probe used (probe a) and the expected sizes of the *Kpn*I restriction fragments are indicated. The primers used to identify homologous integration events are shown (pyrG10 and F10; S4 Table). (B) PCR analysis of *r3b2* transformants. Total DNA isolated from three transformants was amplified with primers shown in (A) to identify homologous integration events (lanes 3, 5 and 7). Arrows mark the size of the expected fragment. As a positive control, the same samples were amplified with internal primers corresponding to the disruption fragment (lanes 2, 4 and 6). M, GeneRuler DNA ladder mixture (Fermentas). (C) Southern blot analysis of the wild-type strain R7B and four *r3b2* transformants. Genomic DNA (1 μg) was digested with *Kpn*I and hybridized with probe shown in (A). Transformants 1, 3 and 4 correspond to those shown in (B). Transformant 2, which did not amplify the expected fragment in the PCR experiment, contains a wild type *r3b2* locus. The positions and sizes of the GeneRuler DNA ladder mixture (M) (Fermentas) size markers are indicated.
(PDF)

S9 Fig Disruption of the 136157 gene. (A) Schematic representation of the wild-type 136157 locus (middle) and after homologous recombination with the disruption fragment (below). Green and blue boxes represent genomic 136157 locus and adjacent sequences, respectively; red boxes, *pyrG* selectable marker; dashed lines, sequences not included in the disruption fragment. The positions of the probes used (probes b and c) and the expected sizes of the *EcoR*I restriction fragments are indicated. The primers used to amplify the disruption fragment from the knockout vector pMAT768 (F2 and R2; see S1 Supporting Information) and to identify homologous integration events (pyrg-R2 and F1) are shown (S4 Table). (B) Southern blot analysis of the wild-type strain R7B and four 136157 transformants. Genomic DNA (1 μg) was digested with *EcoR*I and hybridized with probe b (left) and with probe c (right). M, GeneRuler DNA ladder mixture (Fermentas).
(PDF)

S10 Fig Disruption of the 110239 and 77996 genes. (A) Schematic representation of the wild-type 110239 locus (middle) and after homologous recombination with the disruption fragment (below). Orange and blue boxes represent genomic 110239 locus and adjacent sequences, respectively; red boxes, *pyrG* selectable marker; dashed lines, sequences not included in the disruption fragment. The position of the probe used (probe d) and the expected sizes of the *Sac*I restriction fragments are indicated. The primers used to amplify the disruption fragment from the knockout vector pMAT763 (F5 and R5; see S1 Supporting Information) and to identify homologous integration events (pyrg-F2 and R4) are shown (S4 Table). (B) Similar representation of the 77996 locus in the wild-type and disrupted strains. The positions of the probe used (probes e) and the expected sizes of the *Bgl*II/*Pvu*I restriction fragments are indicated. Primers F8 and R8 were used to amplify the disruption fragment from the knockout vector pMAT770 (see S1 Supporting Information) and primers pyrg-F2 and R7 (S4 Table) were used to identify integration events. (C) Southern blot analysis of the wild-type strain R7B and six 110239 transformants grown in selective medium for ten vegetative cycles. Genomic DNA (1 μg) was digested with *Sac*I and hybridized with probe d, which recognized the wild-type and disrupted alleles but could discriminate between them. **(D)** Southern blot analysis of the wild-type strain R7B and four 77996 transformants grown in selective medium for ten vegetative cycles. Genomic DNA (1 μg) was double digested with *Bgl*II and *Pvu*I and hybridized with probe e, which recognized the wild-type and disrupted alleles but could discriminate between them. The positions and sizes of the GeneRuler DNA ladder mixture (M) (Fermentas) size markers are indicated.
(PDF)

S11 Fig Accumulation of rdRNAs from genes involved in heme B biosynthesis or metabolism in the *r3b2* ^-^ mutant. The heat map shown in Fig 4A is extended to include data from the mutant affected in the RNase gene *r3b2*. Each colored cell in the heat map represents the log_2_ fold change of rdRNAs in the different mutants relative to wild type strain. Expression levels above 0 represent up-accumulation, whereas those below 0 represent down-accumulation. Data were taken from S1 and S6 Tables.
(PDF)

S12 Fig Integration of *r3b2* alleles into the *carRP* locus. (A) Schematic representation of the wild-type *carRP* locus (middle) and after homologous recombination with the disruption fragment (below). The disruption fragment contains the wild type or mutant *r3b2* alleles (*r3b2* ^*wt*^ and r3b2*, respectively) (yellow boxes) and the *leuA* selectable marker (red boxes) flanked by upstream and downstream sequences of the *carRP* gene (blue boxes). Dashed lines indicate sequences not included in the disruption fragment. The position of the probe used (probe f) and the expected sizes of the *Eco*RI restriction fragments are indicated. The primers used to amplify the disruption fragment from the knockout vectors pMAT787 (*r3b2* ^*wt*^) and pMAT788 (r3b2*) (carRP-F1 and carRP-R1; see S1 Supporting Information) are shown (S4 Table). (B) Southern blot analysis of the wild-type strain and transformants of the *r3b2* ^*-*^ mutant strain (Δ*r3b2*) containing the r3b2* mutant allele (transformants 1–5) or *r3b2* ^*wt*^ wild type allele (transformants 5–7) integrated into the *carRP* locus. Genomic DNA (1 μg) was digested with *Eco*RI and hybridized with probe f, which recognized the wild type and disrupted alleles but could discriminate between them. All but one transformants (transformant 4) are homokaryotics for the integration of the *r3b2* alleles into the *carRP* locus.
(PDF)

S13 Fig The Mucoralean R3B2 protein family. The domain organization of proteins identified in the FungiDB genomics database by their similarity to R3B2 is shown. Orange boxes signal the position of the RNase III family signature, with the starting and stopping amino acid indicated. Blue boxes correspond to dsRNA-binding domains. Total number of amino acid residues and the e- value obtained in the BLAST analysis with R3B2 are also shown.
(PDF)

S14 Fig Regulation of the heme group biosynthesis pathway by the non-canonical RNA degradation pathway. (A) Heme B biosynthesis pathway. Some reactions occur in the cytoplasm and some in the mitochondrion (light blue). Substrates and products of each enzymatic step are indicated. The numbers correspond to the enzymatic activities shown in (B). The side branch leading to siroheme synthesis is schematically shown. Steps indicated in red are controlled by enzymes regulated by the *rdrp*-dependent *dicer*-independent degradation pathway. (B) *M*. *circinelloides* proteins corresponding to the enzymatic activities involved in heme biosynthesis pathway shown in (A).
(PDF)

S1 Table Log_2_ fold change and strand bias of *rdrp*-dependent *dicer*-independent sRNAs in different mutants compared to wild type. Normalized reads were used to calculate the fold change of sRNAs in each mutant compared to wild type strain. The sRNA-producing loci are separated into *rdrp-1* and *rdrp-2* dependent (yellow background), only *rdrp-1* dependent (white background) and only *rdrp-2* dependent (blue background). Among each class, data were sorted for the fold change in *rdrp-1* ^*-*^ and/or *rdrp-2* ^*-*^ strains. Values that represent a fourfold or larger change are in bold. Decrease in expression is shown in red and increase in expression is shown in green. The coordinates correspond to the exonic loci where small RNAs map (http://genome.jgi-psf.org/Mucci1/Mucci1.home.html [v1]). Strand bias indicates orientation to mRNAs, where 1 corresponds to all sRNAs in the same orientation as the mRNA, 0 to equal mixture of sRNAs on both strands and -1 to all sRNAs antisense to mRNAs. The fold change of Ago-1 bound sRNAs in the WT compared with the *ago-1* ^*-*^ mutant is shown for the exonic *rdrp*-dependent *dicer*-independent sRNAs identified among those bound to Ago-1. n.d.: sRNAs not detected among those specifically bound to Ago-1. Only loci with a normalized abundance count higher than 50 in the wild type strain and a log_2_ fold change ≥ -2 (fourfold or larger change) were considered.
(XLS)

S2 Table Oligonucleotides used in validation experiments. Sequences of oligonucleotides used to amplify probes for sRNA and mRNA detection of the *rdrp*-dependent *dicer*-independent loci analyzed in Figs 1 and 2, S2 and S3 Figs.
(DOC)

S3 Table Candidate RNase proteins in the *Mucor* genome. Protein ID, coordinates of the corresponding genes in the *M*. *circinelloides* genome (v2), number of amino acid residues and putative domains found in each protein are indicated.
(DOCX)

S4 Table Oligonucleotides used for cloning and functional analysis of the candidate RNase genes. (DOCX)

S5 Table Normalized reads and log_2_ fold change of exonic sRNAs in the *r3b2* ^-^ mutant compared to the wild type. Normalized reads in the wild type strain R7B and the *r3b2* ^*-*^ mutant (WT and *r3b2* ^*-*^ abundance) of exonic sRNAs corresponding to each locus are shown. The coordinates correspond to the exonic loci where small RNAs maps (http://genome.jgi-psf.org/Mucci1/Mucci1.home.html [v1]). Strand bias indicates orientation to mRNAs, where 1 corresponds to all sRNAs in the same orientation as the mRNA, 0 to equal mixture of sRNAs on both strands and -1 to all sRNAs antisense to mRNAs. Normalized reads were used to calculate the fold change of sRNAs in the *r3b2* ^*-*^ mutant compared to wild type strain. The data are sorted for the fold change in *r3b2* ^*-*^ strain. N/A, not applicable. Only loci with a normalized abundance count higher than 50 in the wild type strain and a log_2_ fold change ≥ -2 (fourfold or larger change) were considered.
(XLSX)

S6 Table Log_2_ fold change of the rdRNAs in the *r3b2* ^-^ mutant compared to wild type. The fold change of the *r3b2* ^*-*^ mutant relative to the wild type is shown for the different classes of rdRNAs produced by the non-canonical *rdrp*-dependent *dicer*-independent degradation pathway. Loci that are *rdrp-1* and *rdrp-2* dependent, only *rdrp-1* dependent and only *rdrp-2* dependent have yellow, white and blue background, respectively. Data are sorted as in S1 Table. The log_2_ fold change of rdRNAs in the *rdrp-1* ^*-*^ and *rdrp-2* ^*-*^ mutants compared to the wild type are shown for comparison. Values that represent a fourfold or larger change are in bold. N/A, not applicable.
(XLSX)

S7 Table Log_2_ fold change of the *dicer*-dependent ex-siRNAs in the *r3b2* ^-^ mutant compared to wild type. Data from S5 Table were taken to show the log_2_ fold changes of the different classes of *dicer*-dependent ex-siRNAs in the *r3b2* ^*-*^ mutant. Log_2_ of the fold changes in *dcl* ^*-*^ (*dcl-1* ^*-*^, *dcl-2* ^*-*^ and the double mutant *dcl-1* ^*-*^/*dcl-2* ^*-*^) and *rdrp* ^*-*^ (*rdrp-1* ^*-*^ and *rdrp-2* ^*-*^) mutants [11], as well as the log_2_ fold change of Ago-1-bound siRNAs in the wild type compared with the *ago-1* ^*-*^ mutant [14] are also shown in this table. Data are sorted for the fold change in *dcl-2* ^*-*^ strain, which almost perfectly separated the four ex-siRNA classes from each other. Class I, II, III and IV exons have yellow, white, blue and grey background, respectively. Numbers in bold indicate values that represent a fourfold or larger change. Decrease in expression is shown in red and increase in expression is shown in green. n.d.: ex-siRNAs not detected among those specifically bound to Ago-1. N/A, not applicable.
(XLSX)

S1 Supporting Information Supplementary Materials and Methods. Detailed description of plasmid construction and generation of knock-out mutants.
(DOC)
