# Supplementary figures and images for: A Non-canonical RNA Silencing Pathway Promotes mRNA Degradation in Basal Fungi
Source: PLoS Genet. 2015 Apr 13;11(4):e1005168. doi: 10.1371/journal.pgen.1005168 (PMC4395119; doi:10.1371/journal.pgen.1005168)

S1 Fig.

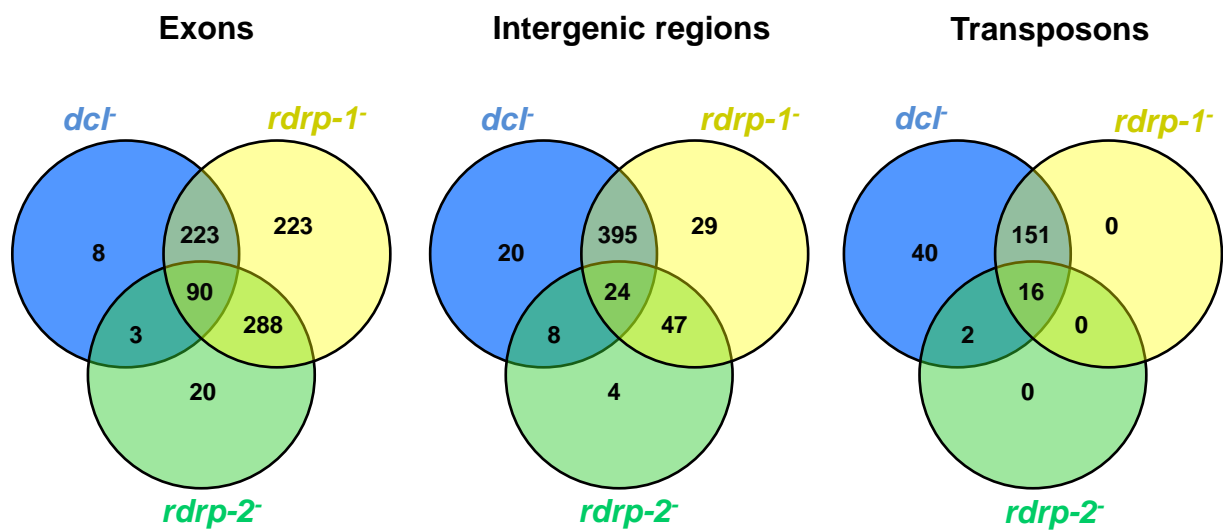

Supplement: S1 Fig — All loci dependent on any of the dicer genes (dcl-1-, dcl-2- or dcl-1/dcl-2-dependent) are included in the same category (dcl -). (PDF) [file pgen.1005168.s001.pdf]

S2 Fig.

Locus 2

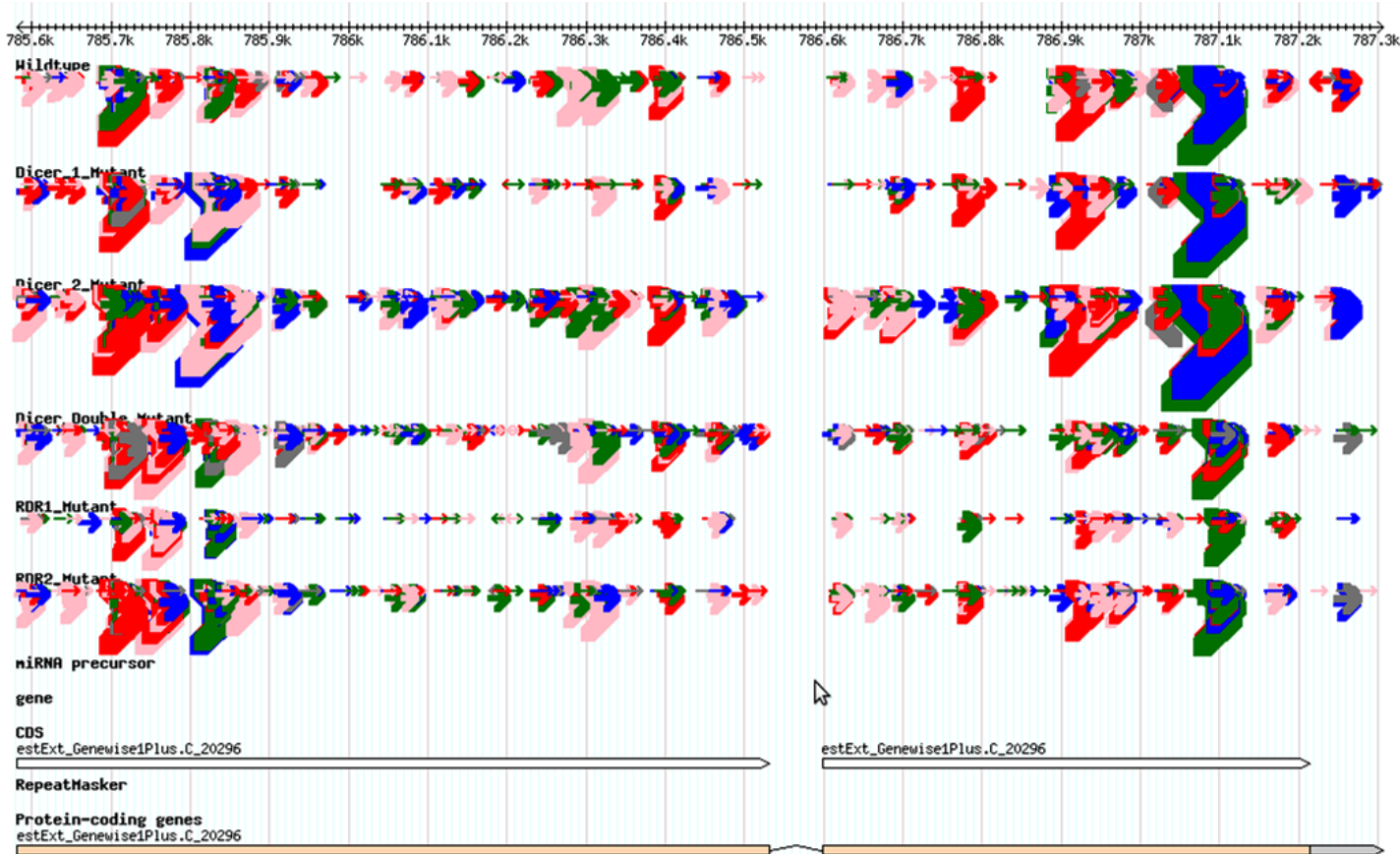

← probe

Locus 3

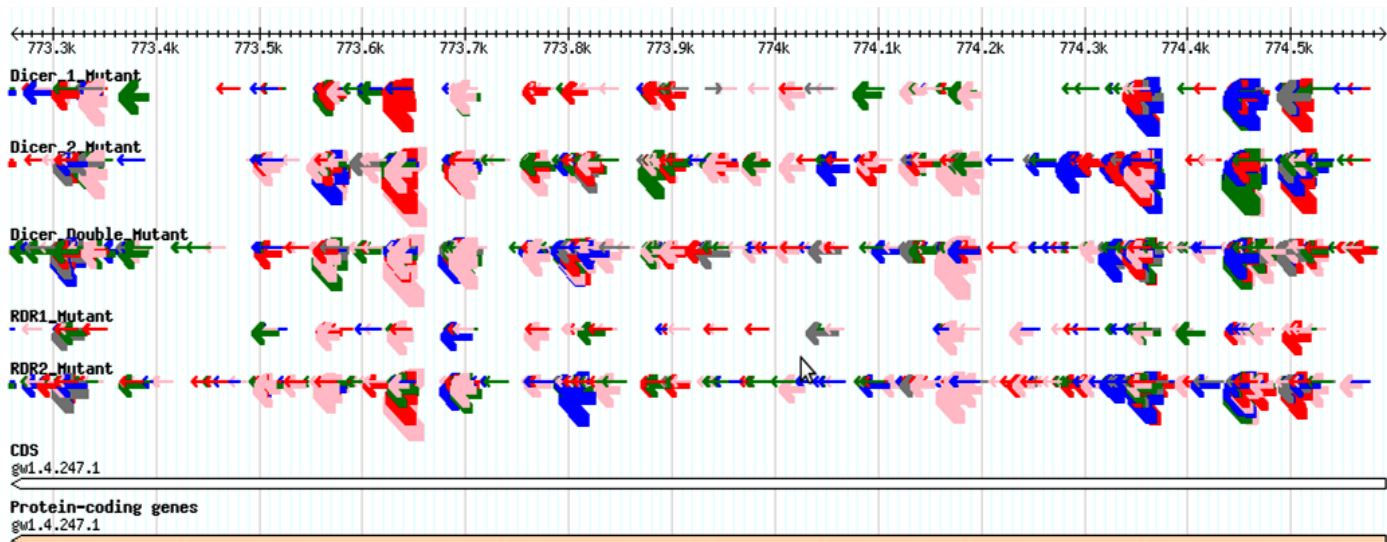

probe

Locus 4

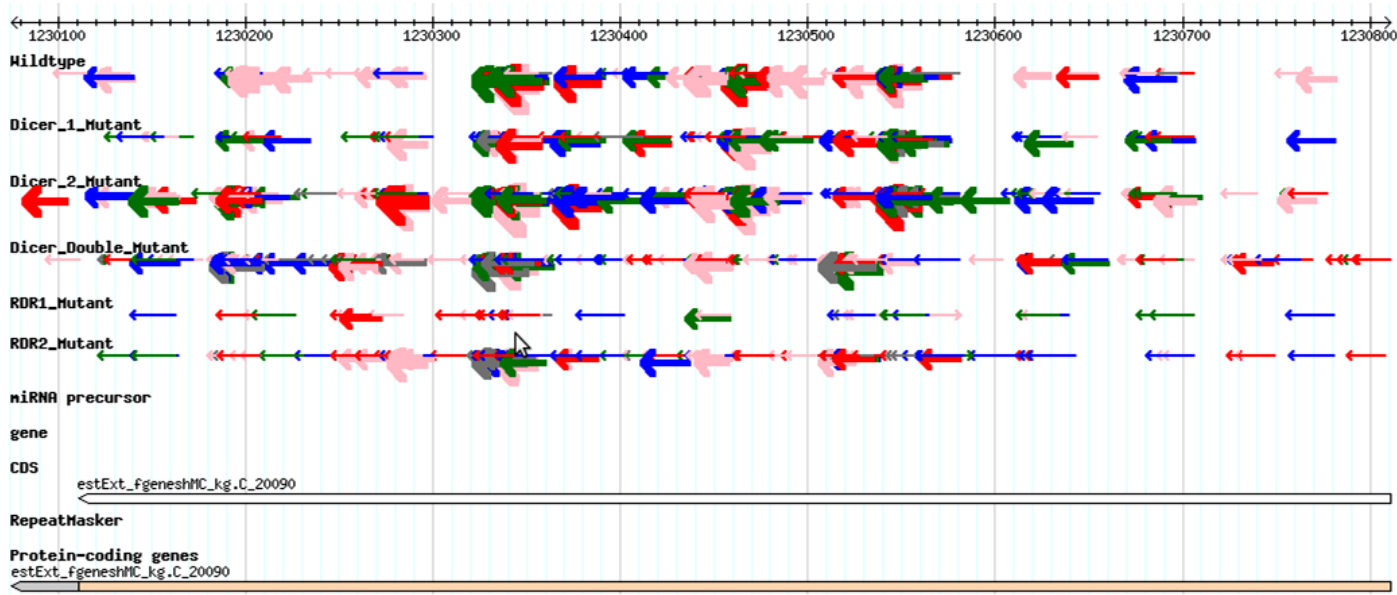

probe

Locus 5

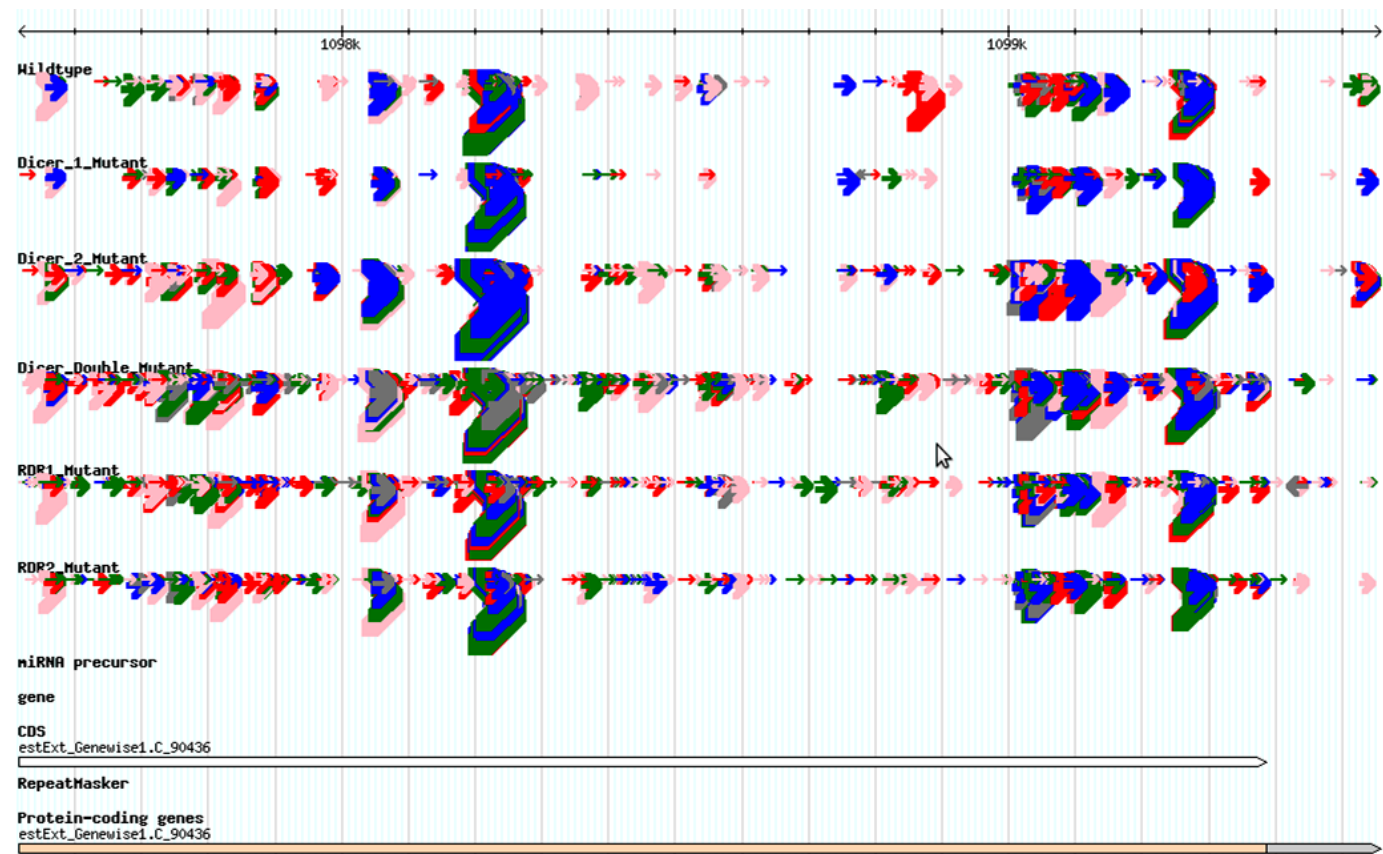

←  
probe

Locus 6

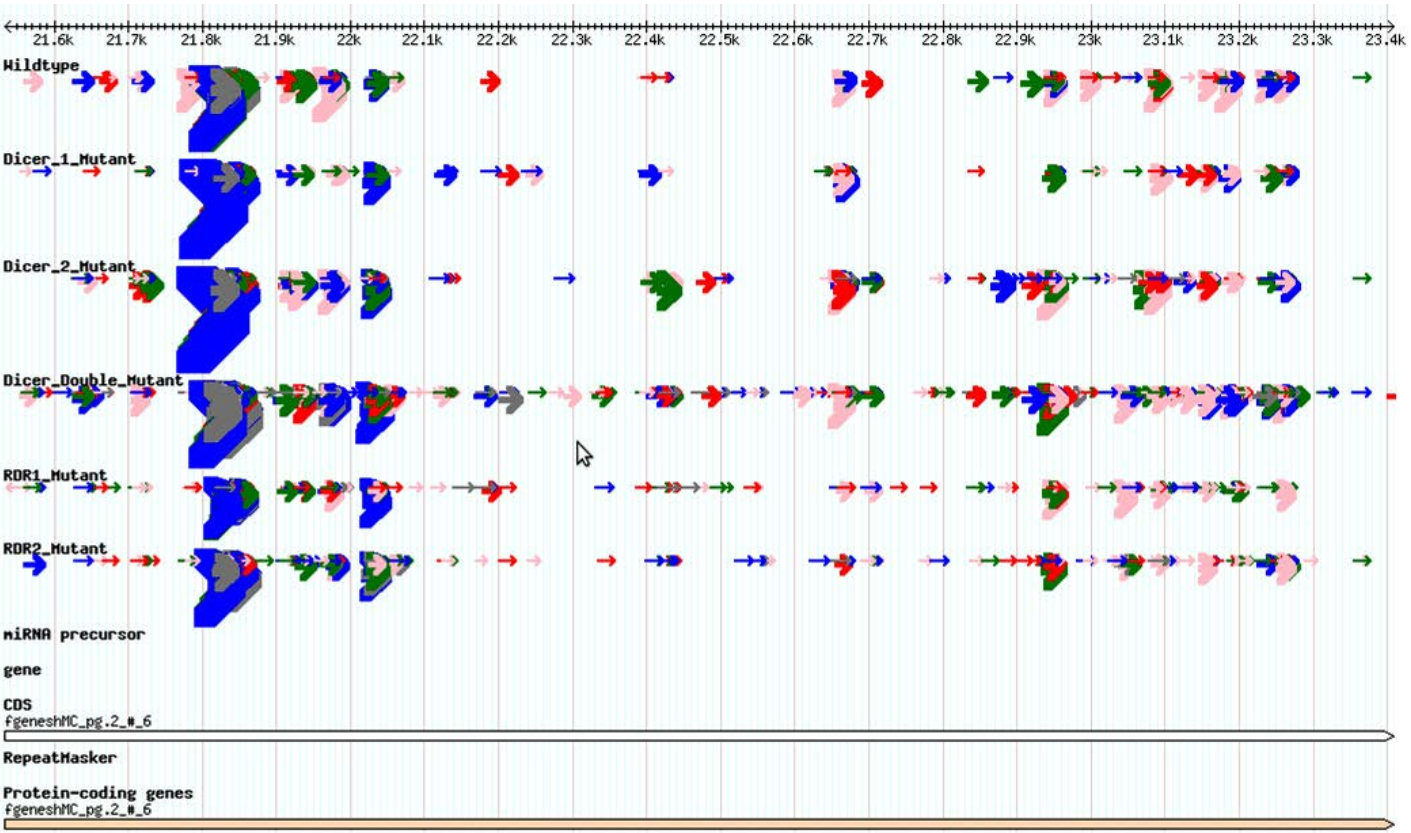

← probe

Locus 7

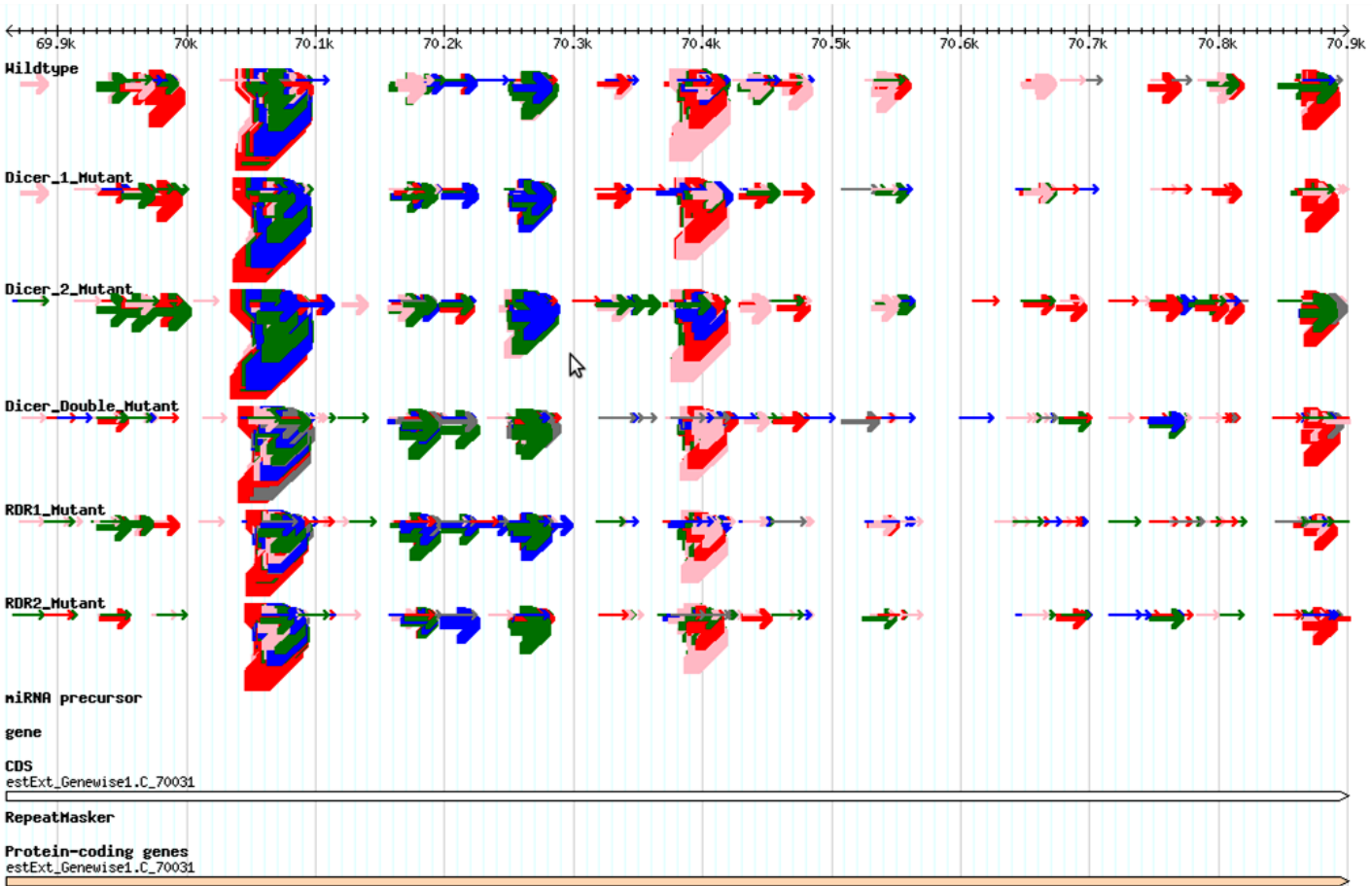

← probe

Supplement: S2 Fig — Several rdrp-dependent dicer-independent sRNA-producing loci were randomly selected to show sRNA accumulation in wt, dcl-1 -, dcl-2 -, dcl-1 -/dcl-2 -, rdrp-1 - and rdrp-2 - strains. Arrows represent sRNA sequence reads (thickness and color references as in Fig 1). Orange arrows represent the position and orientation of probes used for sRNA detection by Northern blots. The exon loci correspond to the following proteins: locus 2: ID 46819, inorganic phosphate transporter; locus 3: ID 11610, unknown protein; locus 4: ID 94060, phosphatase involved in carbohydrate transport and metabolism; locus 5: ID 30368, heat shock protein; locus 6: ID 77714, Dipeptidyl aminopeptidase; locus 7: ID 29487, succinate dehydrogenase, flavoprotein subunit. (PDF) [file pgen.1005168.s002.pdf]

**S3 Fig.**

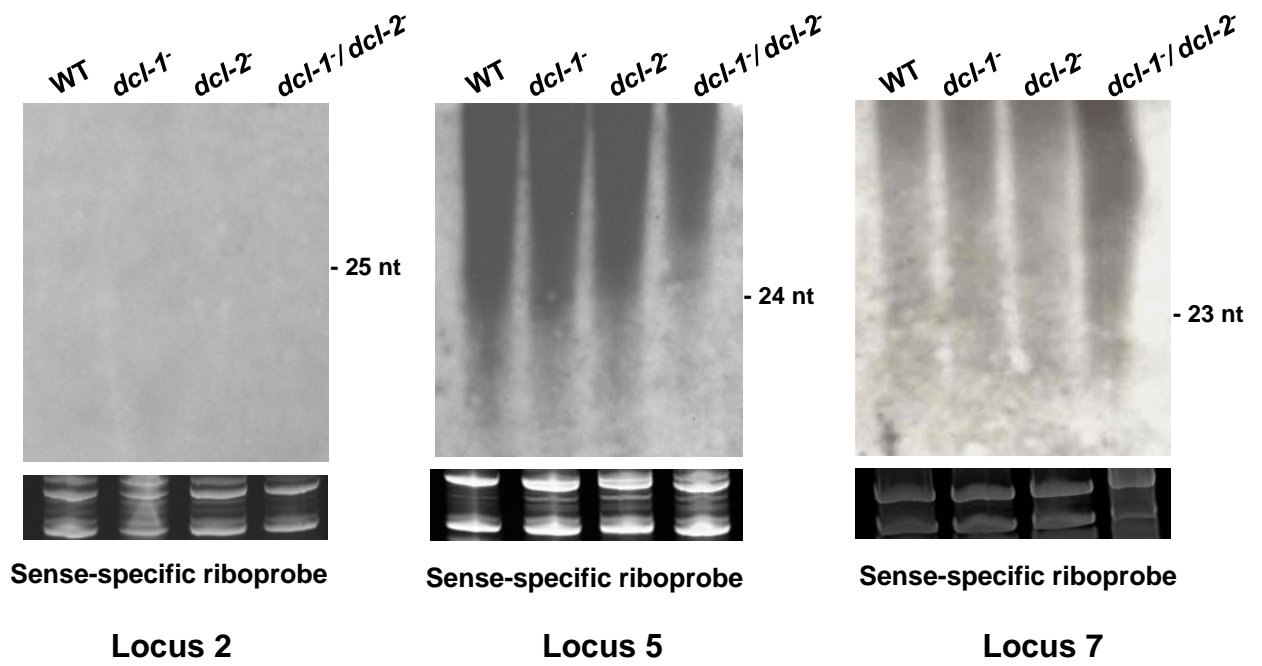

Supplement: S3 Fig — Low-molecular weight RNA (50 μg) was extracted from wild-type, dcl-1 -, dcl-2 - and dcl-1 - /dcl-2 - mutant strains and probed with sense and antisense-specific riboprobes specific to each locus (S2 Table; see S2 Fig). Ethidium bromide stained images of gels below the radiograms show equal loading of lanes. Ten picomoles per lane of 23-mer to 25-mer DNA oligonucleotides in antisense and sense orientation were used as size markers and to control the hybridization specificity. In all cases, the RNA probes hybridized to these controls. Results obtained for three representative loci are shown (see S2 Fig). The antisense-specific riboprobes did not give any signal in any of the loci analyzed. (PDF) [file pgen.1005168.s003.pdf]

S4 Fig.

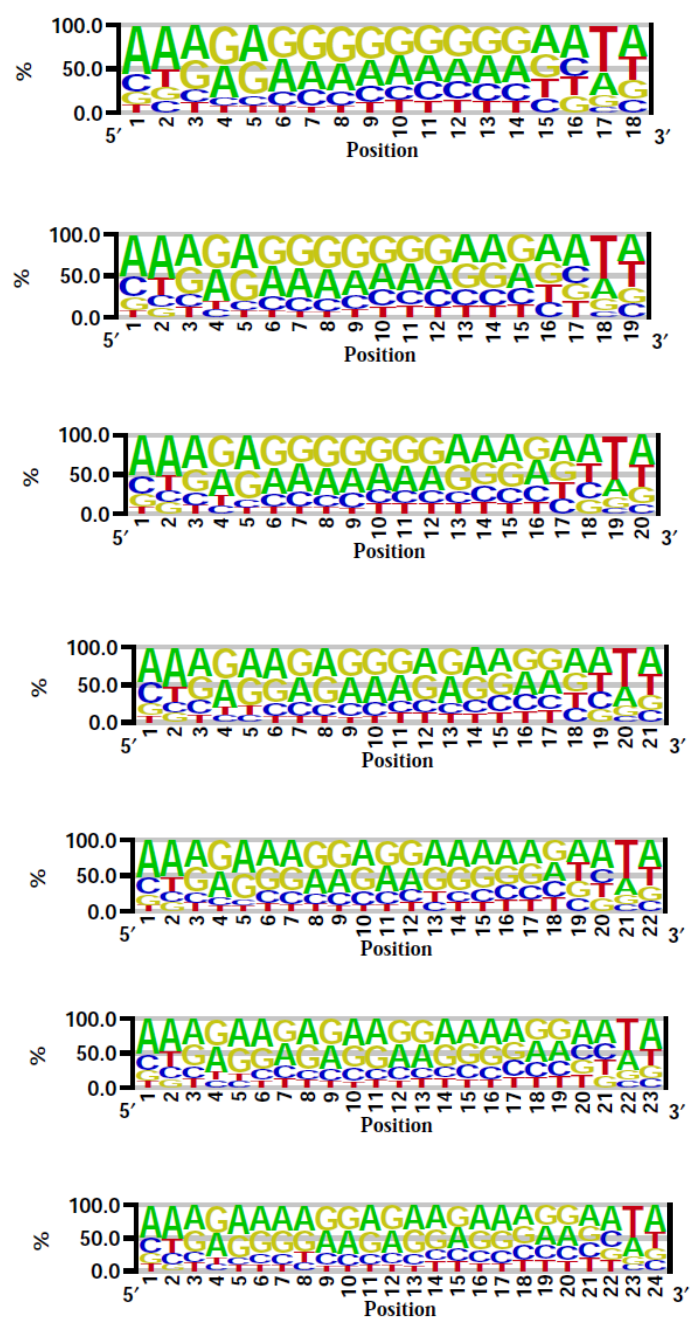

Supplement: S4 Fig — The frequency of each of the four bases was calculated in rdrp-dependent dicer-independent exonic sRNAs. The result is shown for separate sizes of 18–24 nts. The numbers on the x-axis refer to the position in the sRNAs and the y-axis shows the percentage distribution. The top to bottom order of bases in each position is determined by their frequency (highest on top). The figure shows that uracil (T in the cDNA sequences) is highly enriched at the penultimate position and under-represented in the rest of the sRNA sequence. (PDF) [file pgen.1005168.s004.pdf]

S5 Fig.

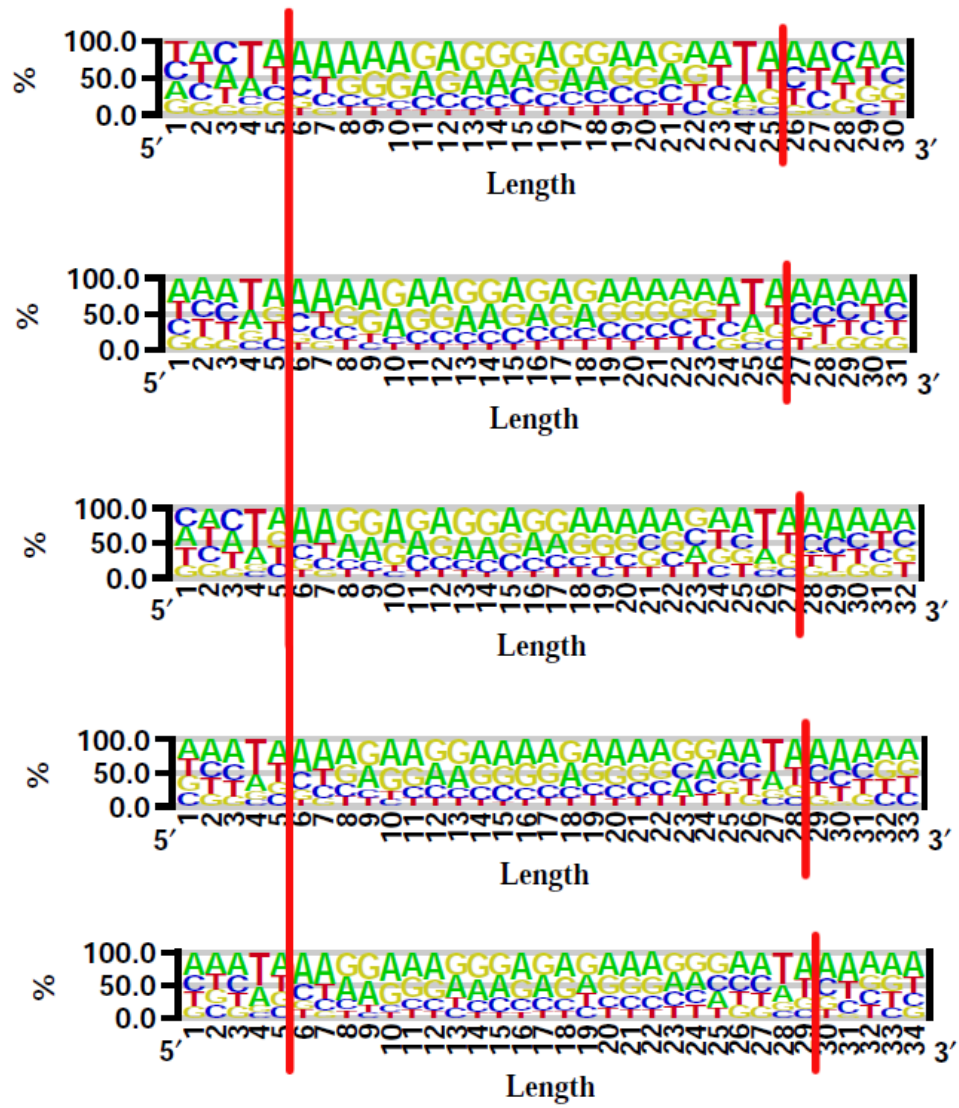

Supplement: S5 Fig — The frequency of each of the four bases was calculated 5-bp upstream and 5-bp downstream of the sRNA. The result is shown for separate sizes of 20–24 nts. Red lines mark the ends of the sRNA sequence. The numbers on the x-axis refer to the position in the genomic sequence and the y-axis shows the percentage distribution. The top to bottom order of bases in each position is determined by their frequency (highest on top). The figure shows that uracil (T in the cDNA sequences) is highly over-represented in the position -2 relative to the sRNA 5’-end. (PDF) [file pgen.1005168.s005.pdf]

S6 Fig.

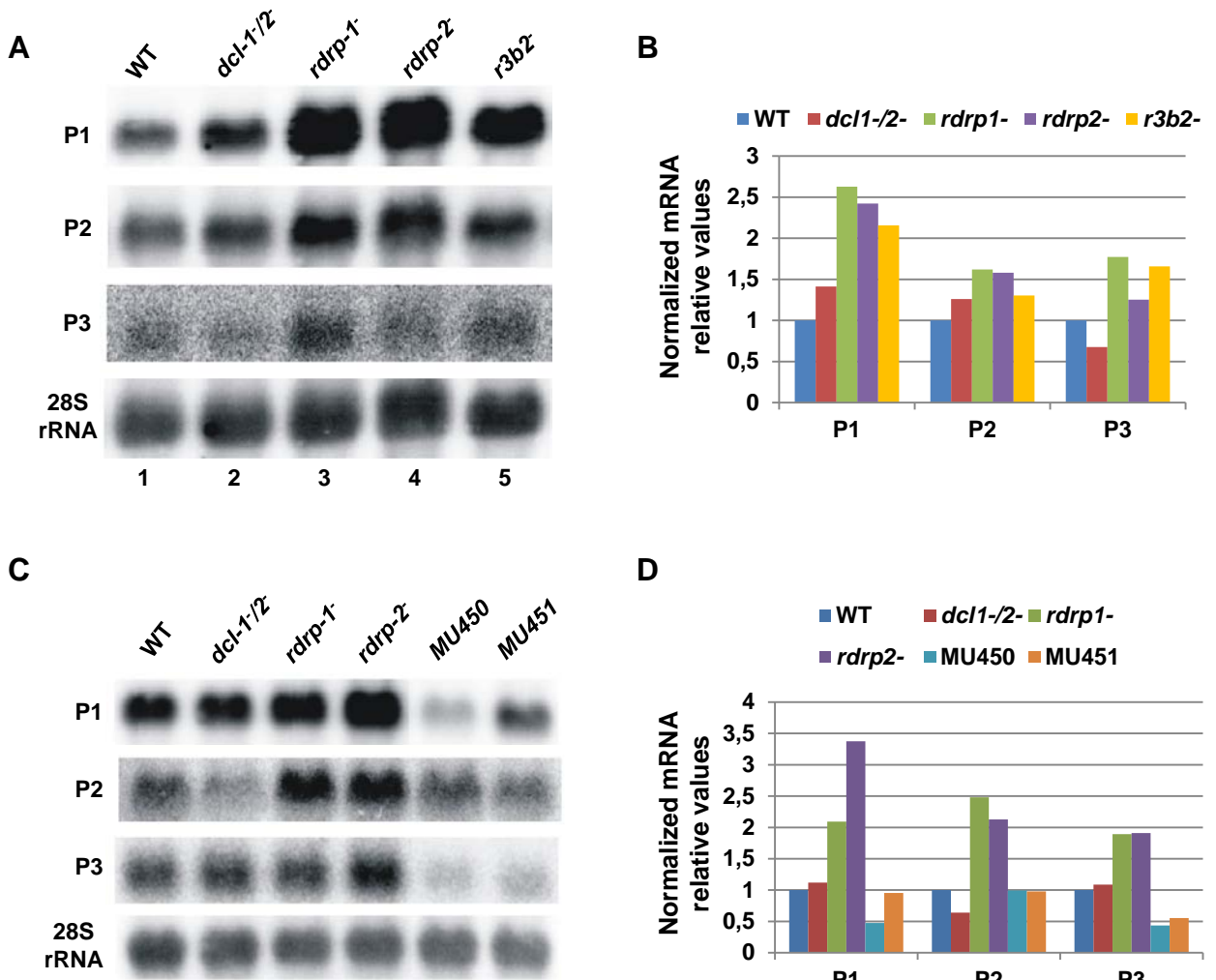

Supplement: S6 Fig — (A) Northern blots of high molecular weight RNAs corresponding to rdRNA-producing exons (genes P1 to P3) were carried out using total RNA (50 μg) extracted from wild type, dcl - and rdrp - mutant strains (lanes 1–4) and a mutant affected in the ribonuclease gene r3b2 (lane 5) grown for 48 hours in MMC medium. Samples were separated in 1.2% denaturing agarose gel, transferred to membranes and hybridized with gene specific probes (S2 Table). Genes P1 to P3 correspond to those indicated in Fig 2. The membranes were reprobed with a 28S rRNA probe to check loading. Images are representative of two independent experiments (B) Densitometric analysis of expression data shown in (A). Signal intensities were quantified and normalized to rRNA levels. All data were again normalized with respect to the expression value of the wild type strain (R7B) for each gene. (C) Accumulation of mRNA from the rdRNA-producing exons (P1 to P3) in mutants affected in the 136157 RNase. Total RNA (50 μg) extracted from the MU450 and MU451 mutants, affected in the 136157 RNase gene, as well as the wild type strain and dcl - and rdrp - mutants grown for 24 hours in liquid MMC medium was hybridized with gene specific probes (S2 Table). The membrane was reprobed with a 28S rRNA probe as loading control. Images are representative of two independent experiments. (D) Densitometric analysis of expression data shown in (C). Signal intensities were quantified and normalized as in (B). (PDF) [file pgen.1005168.s006.pdf]

S7 Fig.

A

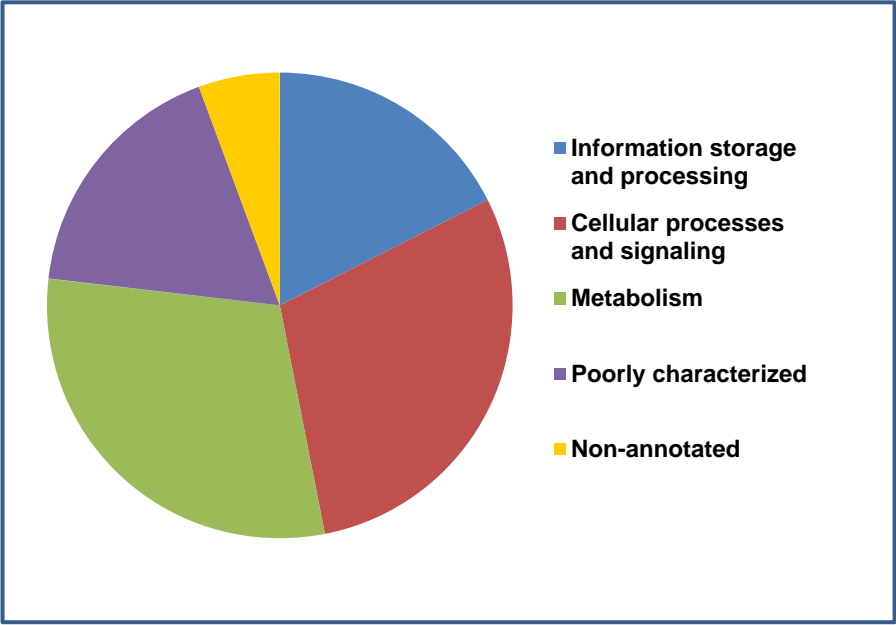

B

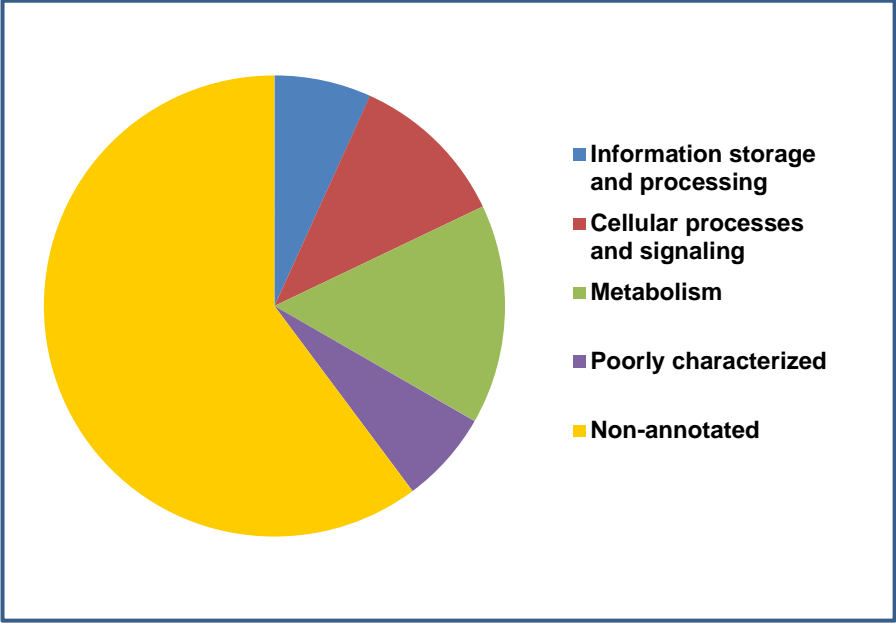

Supplement: S7 Fig — (A) The proportion of genes regulated by the rdrp-dependent dicer-independent non-canonical pathway within the different KOG biological process categories is shown. Data were taken from S1 Table. (B) Similar analysis of genes regulated by canonical dicer-dependent ex-siRNAs is shown for comparison. Data were taken from [11]. (PDF) [file pgen.1005168.s007.pdf]

S8 Fig.

A

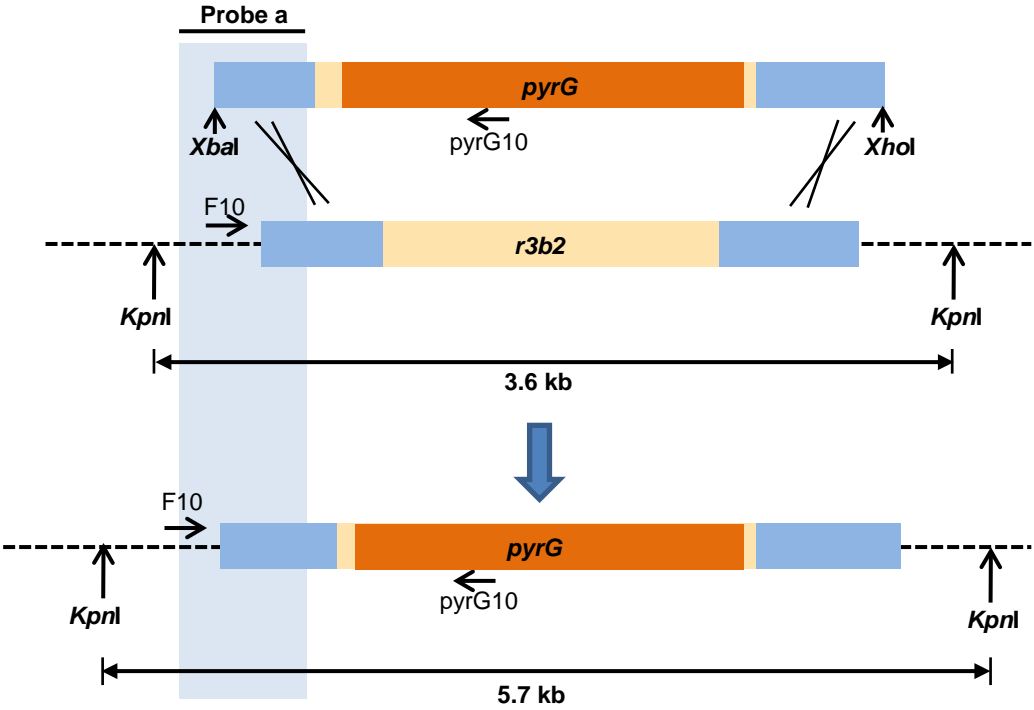

B

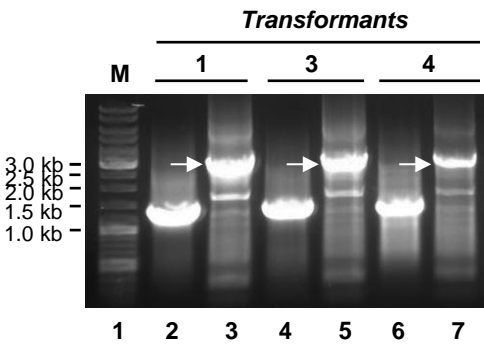

C

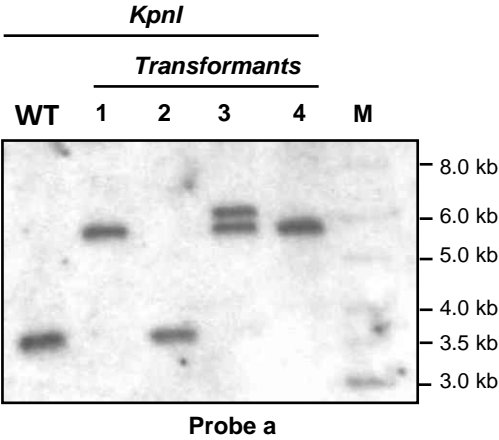

Supplement: S8 Fig — (A) Schematic representation of the wild-type r3b2 locus (middle) and after homologous recombination with the disruption fragment (below). Yellow and blue boxes represent genomic r3b2 locus and adjacent sequences, respectively; red boxes, pyrG selectable marker; dashed lines, sequences not included in the disruption fragment. The XbaI and XhoI sites used to release the disruption fragment from the knockout vector pMAT1298 are indicated (see S1 Supporting Information). The positions of the probe used (probe a) and the expected sizes of the KpnI restriction fragments are indicated. The primers used to identify homologous integration events are shown (pyrG10 and F10; S4 Table). (B) PCR analysis of r3b2 transformants. Total DNA isolated from three transformants was amplified with primers shown in (A) to identify homologous integration events (lanes 3, 5 and 7). Arrows mark the size of the expected fragment. As a positive control, the same samples were amplified with internal primers corresponding to the disruption fragment (lanes 2, 4 and 6). M, GeneRuler DNA ladder mixture (Fermentas). (C) Southern blot analysis of the wild-type strain R7B and four r3b2 transformants. Genomic DNA (1 μg) was digested with KpnI and hybridized with probe shown in (A). Transformants 1, 3 and 4 correspond to those shown in (B). Transformant 2, which did not amplify the expected fragment in the PCR experiment, contains a wild type r3b2 locus. The positions and sizes of the GeneRuler DNA ladder mixture (M) (Fermentas) size markers are indicated. (PDF) [file pgen.1005168.s008.pdf]

S9 Fig.

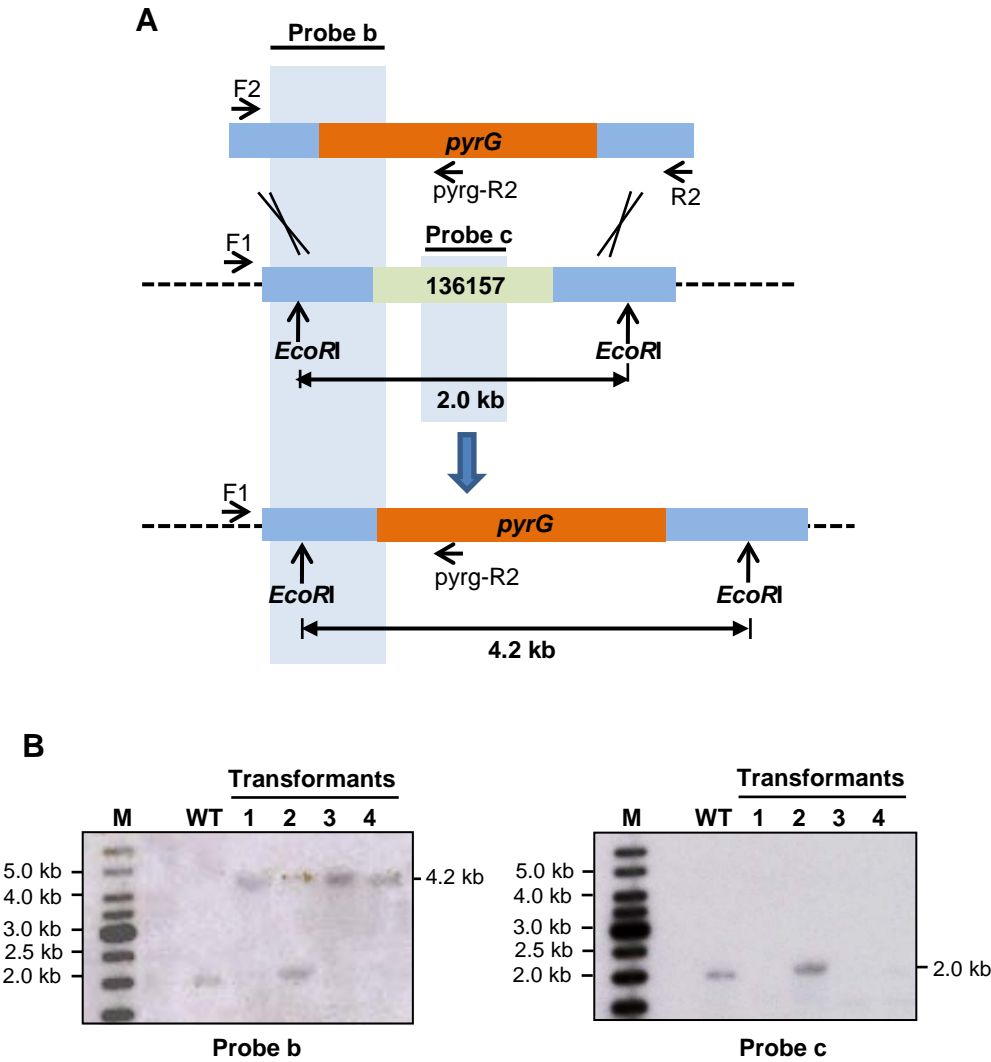

Supplement: S9 Fig — (A) Schematic representation of the wild-type 136157 locus (middle) and after homologous recombination with the disruption fragment (below). Green and blue boxes represent genomic 136157 locus and adjacent sequences, respectively; red boxes, pyrG selectable marker; dashed lines, sequences not included in the disruption fragment. The positions of the probes used (probes b and c) and the expected sizes of the EcoRI restriction fragments are indicated. The primers used to amplify the disruption fragment from the knockout vector pMAT768 (F2 and R2; see S1 Supporting Information) and to identify homologous integration events (pyrg-R2 and F1) are shown (S4 Table). (B) Southern blot analysis of the wild-type strain R7B and four 136157 transformants. Genomic DNA (1 μg) was digested with EcoRI and hybridized with probe b (left) and with probe c (right). M, GeneRuler DNA ladder mixture (Fermentas). (PDF) [file pgen.1005168.s009.pdf]

S10 Fig.

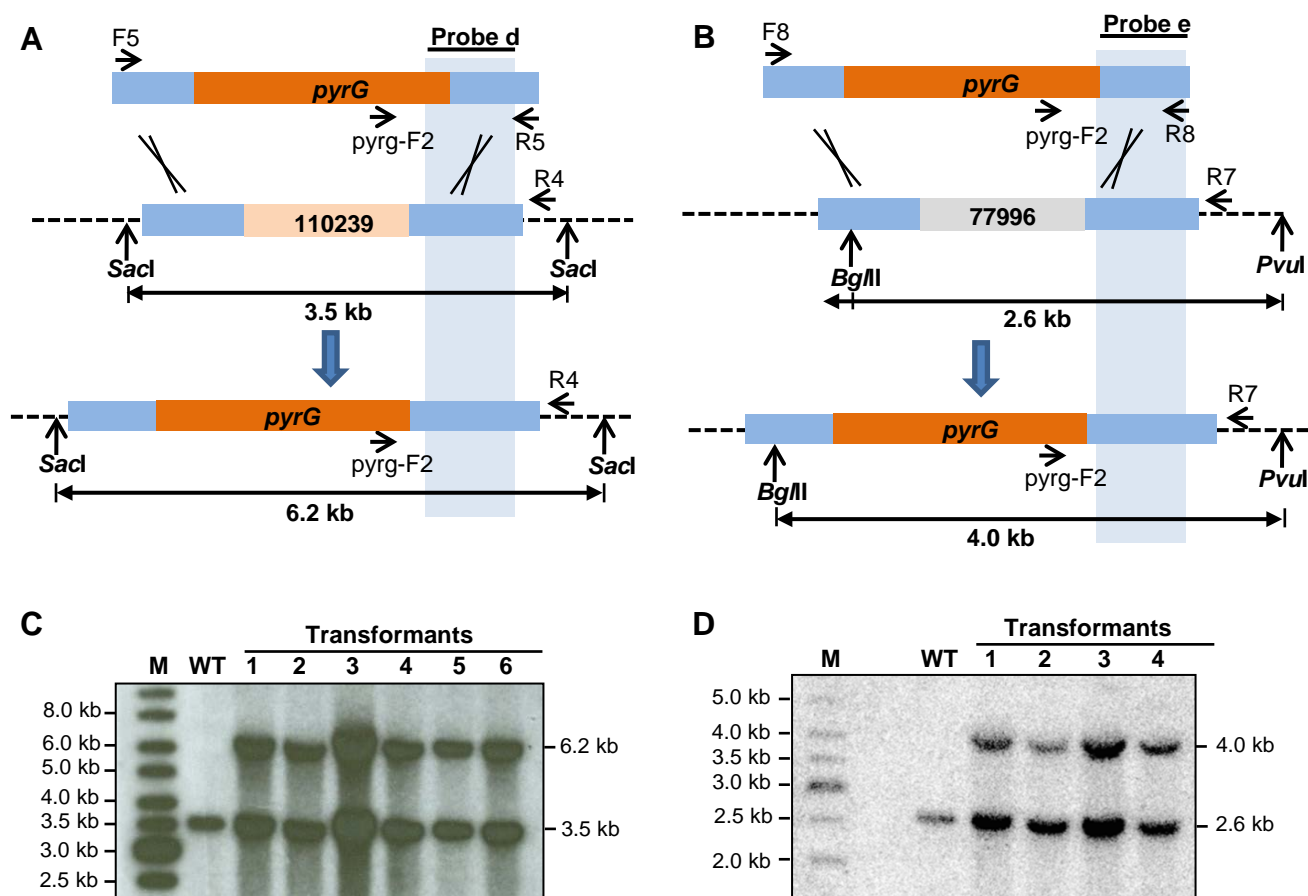

Supplement: S10 Fig — (A) Schematic representation of the wild-type 110239 locus (middle) and after homologous recombination with the disruption fragment (below). Orange and blue boxes represent genomic 110239 locus and adjacent sequences, respectively; red boxes, pyrG selectable marker; dashed lines, sequences not included in the disruption fragment. The position of the probe used (probe d) and the expected sizes of the SacI restriction fragments are indicated. The primers used to amplify the disruption fragment from the knockout vector pMAT763 (F5 and R5; see S1 Supporting Information) and to identify homologous integration events (pyrg-F2 and R4) are shown (S4 Table). (B) Similar representation of the 77996 locus in the wild-type and disrupted strains. The positions of the probe used (probes e) and the expected sizes of the BglII/PvuI restriction fragments are indicated. Primers F8 and R8 were used to amplify the disruption fragment from the knockout vector pMAT770 (see S1 Supporting Information) and primers pyrg-F2 and R7 (S4 Table) were used to identify integration events. (C) Southern blot analysis of the wild-type strain R7B and six 110239 transformants grown in selective medium for ten vegetative cycles. Genomic DNA (1 μg) was digested with SacI and hybridized with probe d, which recognized the wild-type and disrupted alleles but could discriminate between them. (D) Southern blot analysis of the wild-type strain R7B and four 77996 transformants grown in selective medium for ten vegetative cycles. Genomic DNA (1 μg) was double digested with BglII and PvuI and hybridized with probe e, which recognized the wild-type and disrupted alleles but could discriminate between them. The positions and sizes of the GeneRuler DNA ladder mixture (M) (Fermentas) size markers are indicated. (PDF) [file pgen.1005168.s010.pdf]

S11 Fig.

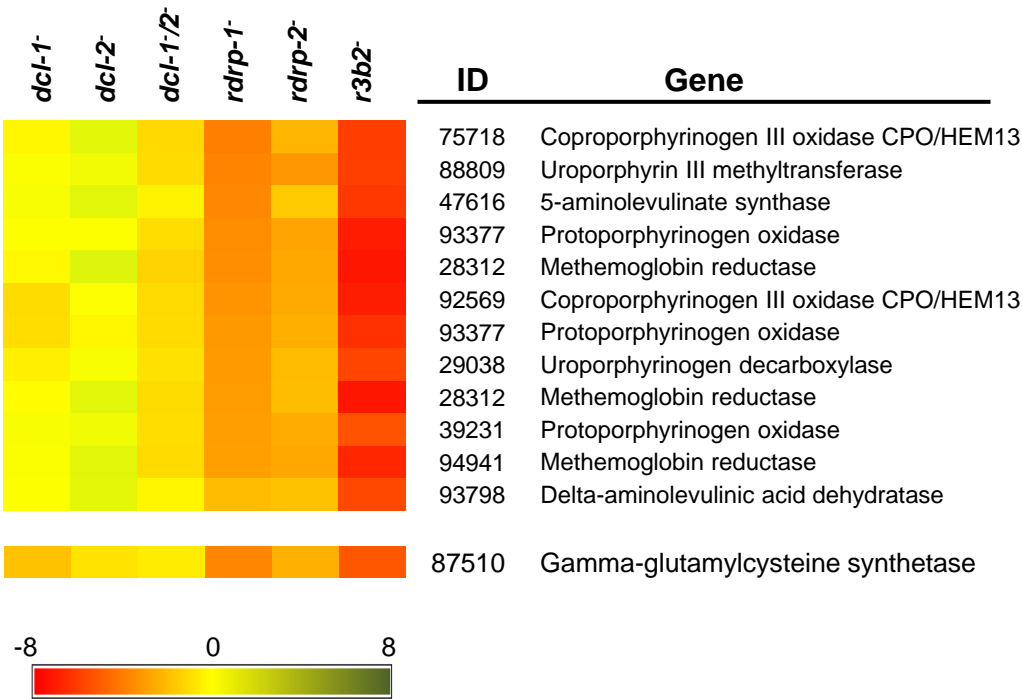

Supplement: S11 Fig — The heat map shown in Fig 4A is extended to include data from the mutant affected in the RNase gene r3b2. Each colored cell in the heat map represents the log2 fold change of rdRNAs in the different mutants relative to wild type strain. Expression levels above 0 represent up-accumulation, whereas those below 0 represent down-accumulation. Data were taken from S1 and S6 Tables. (PDF) [file pgen.1005168.s011.pdf]

S12 Fig.

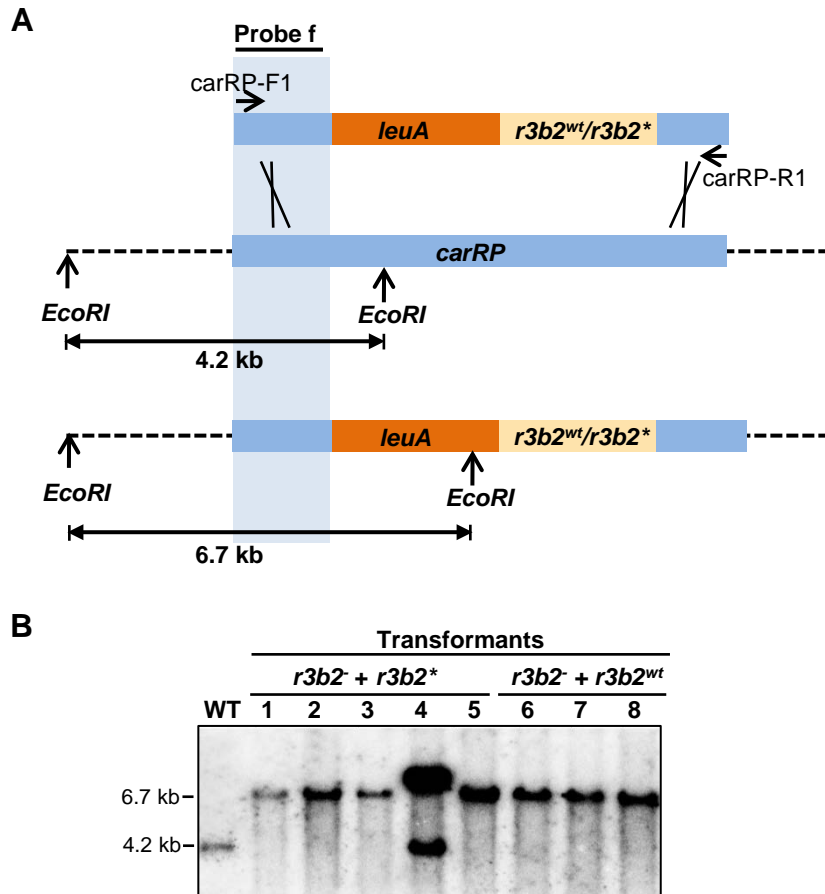

Supplement: S12 Fig — (A) Schematic representation of the wild-type carRP locus (middle) and after homologous recombination with the disruption fragment (below). The disruption fragment contains the wild type or mutant r3b2 alleles (r3b2 wt and r3b2*, respectively) (yellow boxes) and the leuA selectable marker (red boxes) flanked by upstream and downstream sequences of the carRP gene (blue boxes). Dashed lines indicate sequences not included in the disruption fragment. The position of the probe used (probe f) and the expected sizes of the EcoRI restriction fragments are indicated. The primers used to amplify the disruption fragment from the knockout vectors pMAT787 (r3b2 wt) and pMAT788 (r3b2*) (carRP-F1 and carRP-R1; see S1 Supporting Information) are shown (S4 Table). (B) Southern blot analysis of the wild-type strain and transformants of the r3b2 - mutant strain (Δr3b2) containing the r3b2* mutant allele (transformants 1–5) or r3b2 wt wild type allele (transformants 5–7) integrated into the carRP locus. Genomic DNA (1 μg) was digested with EcoRI and hybridized with probe f, which recognized the wild type and disrupted alleles but could discriminate between them. All but one transformants (transformant 4) are homokaryotics for the integration of the r3b2 alleles into the carRP locus. (PDF) [file pgen.1005168.s012.pdf]

S13 Fig.

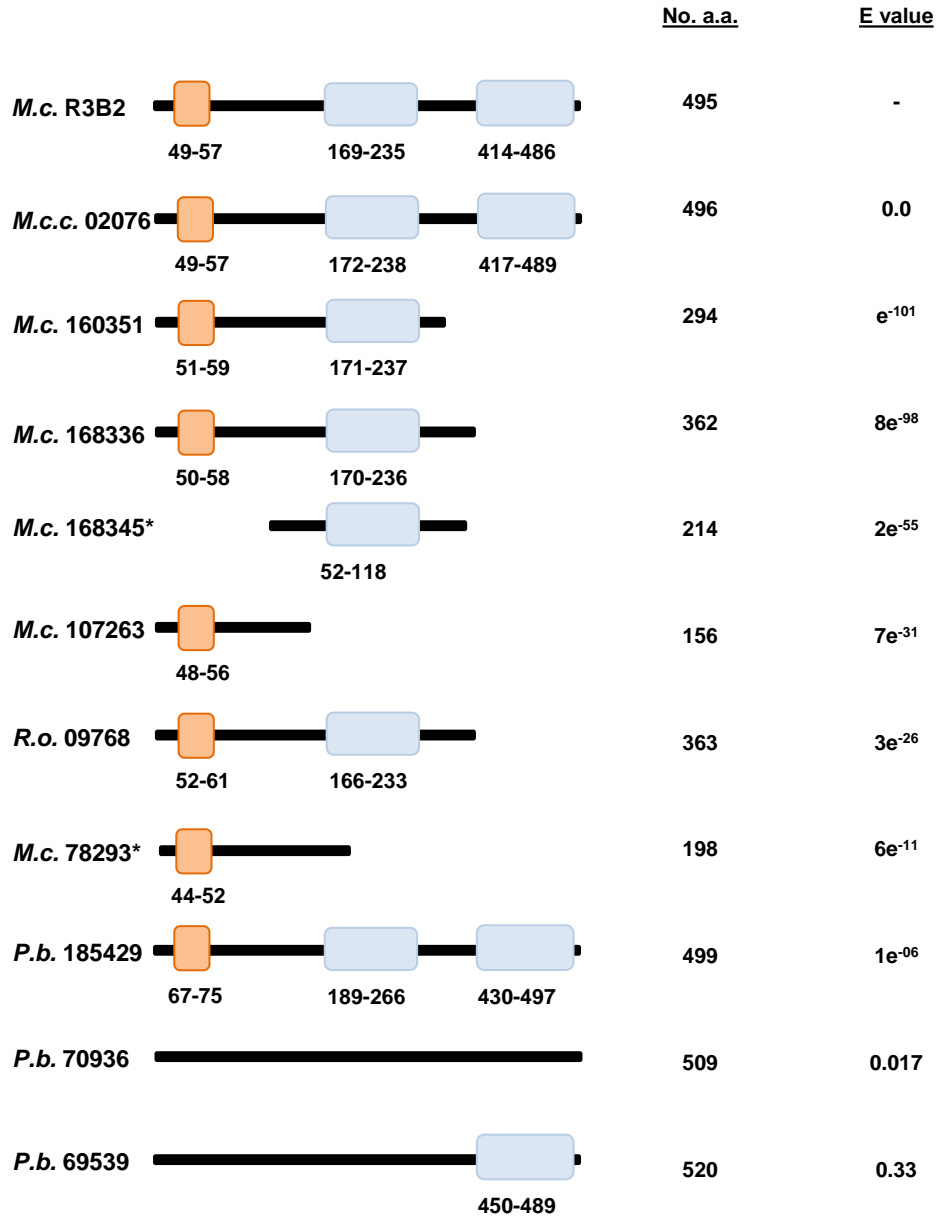

Supplement: S13 Fig — The domain organization of proteins identified in the FungiDB genomics database by their similarity to R3B2 is shown. Orange boxes signal the position of the RNase III family signature, with the starting and stopping amino acid indicated. Blue boxes correspond to dsRNA-binding domains. Total number of amino acid residues and the e- value obtained in the BLAST analysis with R3B2 are also shown. (PDF) [file pgen.1005168.s013.pdf]
