# Supplementary material for: A Non-canonical RNA Silencing Pathway Promotes mRNA Degradation in Basal Fungi
Source: PLoS Genet. 2015 Apr 13;11(4):e1005168. doi: 10.1371/journal.pgen.1005168 (PMC4395119; doi:10.1371/journal.pgen.1005168)
Supplement: S14 Fig — (A) Heme B biosynthesis pathway. Some reactions occur in the cytoplasm and some in the mitochondrion (light blue). Substrates and products of each enzymatic step are indicated. The numbers correspond to the enzymatic activities shown in (B). The side branch leading to siroheme synthesis is schematically shown. Steps indicated in red are controlled by enzymes regulated by the rdrp-dependent dicer-independent degradation pathway. (B) M. circinelloides proteins corresponding to the enzymatic activities involved in heme biosynthesis pathway shown in (A). (PDF) [file pgen.1005168.s014.pdf]

S14 Fig.

A

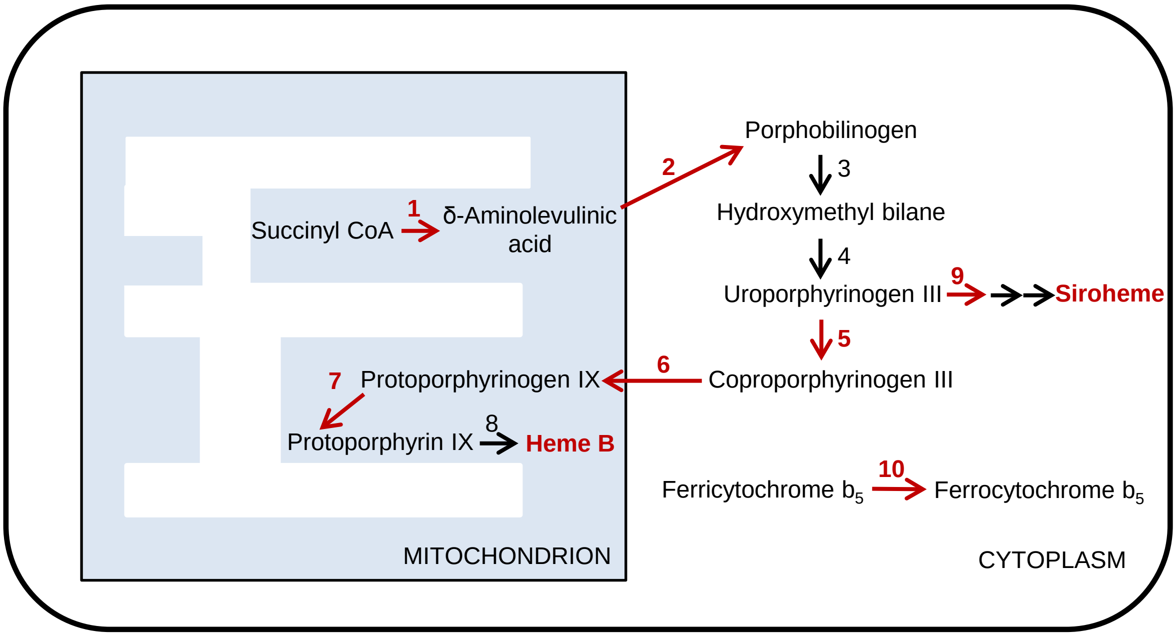

B

No.	Enzyme	*Mucor* protein
1	ALA synthase	ID 47616
2	ALA dehydratase	ID 93798
3	PGB deaminase	
4	Uroporphyrinogen III synthase	
5	Uroporphyrinogen III decarboxylase	ID 29038
6	Coproporphyrinogen III oxidase	ID 75718, ID 92569
7	Protoporphyrinogen oxidase	ID 93377, ID 39231
8	Ferrochelatase	
9	Uroporphyrinogen III methyltransferase	ID 88809
10	Methemoglobin reductase	ID 28312, ID 94941
