# Supplementary material for: A Non-canonical RNA Silencing Pathway Promotes mRNA Degradation in Basal Fungi
Source: PLoS Genet. 2015 Apr 13;11(4):e1005168. doi: 10.1371/journal.pgen.1005168 (PMC4395119; doi:10.1371/journal.pgen.1005168)
Supplement: S2 Table — Sequences of oligonucleotides used to amplify probes for sRNA and mRNA detection of the rdrp-dependent dicer-independent loci analyzed in Figs 1 and 2, S2 and S3 Figs. (DOC) [file pgen.1005168.s016.doc]

**Table S2**. Oligonucleotides used in validation experiments.

| **Name** | **Sequence** | **Use^1^** |
| --- | --- | --- |
| Locus 1 forward | 5’ CCAGTTGTCAGGGAAACCATCC 3’ | Primer for sense and antisense-specific riboprobes for locus 1 sRNAs |
| Locus 1 reverse | 5’ TGTTGGTAACAAGGCTGGTCCTG 3’ | Primer for sense and antisense-specific riboprobes for locus 1 sRNAs |
| Locus 2 forward | 5’ AGAGCCAGATGAACTAATGC 3’ | Primer for sense and antisense-specific riboprobes for locus 2 sRNAs |
| Locus 2 reverse | 5’ TGCTCATCATGGGAACTTGC 3’ | Primer for sense and antisense-specific riboprobes for locus 2 sRNAs |
| Locus 3 forward | 5’ AGGCATTGATCAGATTGGAG 3’ | Primer for sense and antisense-specific riboprobes for locus 3 sRNAs |
| Locus 3 reverse | 5’ AACTTGCTCTACCAACCTTGCTC 3’ | Primer for sense and antisense-specific riboprobes for locus 3 sRNAs |
| Locus 4 forward | 5’ TGATCAATCACACCACCCAACGTG 3’ | Primer for sense and antisense-specific riboprobes for locus 4 sRNAs |
| Locus 4 reverse | 5’ ATTCGACAACTCACTCATGTACTCC 3’ | Primer for sense and antisense-specific riboprobes for locus 4 sRNAs |
| Locus 5 forward | 5’ GAACGTTTGATCGGTGATGCC 3’ | Primer for sense and antisense-specific riboprobes for locus 5 sRNAs |
| Locus 5 reverse | 5’ GAGGTCCTTGGTAATGTCTTCACCG 3’ | Primer for sense and antisense-specific riboprobes for locus 5 sRNAs |
| Locus 6 forward | 5’ GGCAAAGCAAGCTACCGTCTCTCC 3’ | Primer for sense and antisense-specific riboprobes for locus 6 sRNAs |
| Locus 6 reverse | 5’ GGTTGCGGTCAGCTTCGTACTGGG 3’ | Primer for sense and antisense-specific riboprobes for locus 6 sRNAs |
| Locus 7 forward | 5’ CATGTTCCAGGCAACTATCCC 3’ | Primer for sense and antisense-specific riboprobes for locus 7 sRNAs |
| Locus 7 reverse | 5’ CAGTGATAAGGCAACCAGAGCC 3’ | Primer for sense and antisense-specific riboprobes for locus 7 sRNAs |
| P1 forward | 5’ AATGCCAACGAATTGAACGCCTCTTATGCT 3’ | Primer for DNA probe for mRNA from gene P1 |
| P1 reverse | 5’ ACGAGGCATGATGACTTCGATAAAGTGC 3’ | Primer for DNA probe for mRNA from gene P1 |
| P2 forward | 5’ AGACCGAGATTCCCAACATTGCTGCCAT 3’ | Primer for DNA probe for mRNA from gene P2 |
| P2 reverse | 5’ CAAACTTGAGAACGCCTTGGTTTTCCAGAA 3’ | Primer for DNA probe for mRNA from gene P2 |
| P3 forward | 5’ ATGAGTACAGGATTCTCGAGCGCACA 3’ | Primer for DNA probe for mRNA from gene P3 |
| P3 reverse | 5’ CTCATCACACACTTAAATAGAGGAGGCAG 3’ | Primer for DNA probe for mRNA from gene P3 |

^1^ Primers for sense and antisense-specific riboprobes for sRNA validations of the different loci amplified fragments ranging from 500 to 1000 nt. Those fragments were cloned, in different orientations relative to the T7 promoter, into pGEM-T (Promega). Primers for DNA probes for mRNA analysis amplified 1479 bp (P1), 761 bp (P2) and 309 bp (P3) fragments.
