# Supplementary material for: A Non-canonical RNA Silencing Pathway Promotes mRNA Degradation in Basal Fungi
Source: PLoS Genet. 2015 Apr 13;11(4):e1005168. doi: 10.1371/journal.pgen.1005168 (PMC4395119; doi:10.1371/journal.pgen.1005168)
Supplement: S3 Table — Protein ID, coordinates of the corresponding genes in the M. circinelloides genome (v2), number of amino acid residues and putative domains found in each protein are indicated. (DOCX) [file pgen.1005168.s017.docx]

**Table S3**. **Candidate RNase proteins in the *Mucor* genome v2.0.**

| **Protein ID** | **Scaffold** | **Number of residues** | **Domains^1^** |
| --- | --- | --- | --- |
| **77996** | scaffold_02:4034471-4035091 | 166 | RNase III |
| **80729** | scaffold_03:4859492-4861037 | 494 | RNase III-like  2 x dsRBD |
| **110239** | scaffold_04:2084271-2085274 | 297 | RNase III  dsRBD |
| **136157** | scaffold_01:4095479-4096928 | 425 | PKc  RNase_Ire1 |

^1^ RNase III: double-stranded RNA-specific ribonuclease; dsRBD: double stranded RNA binding domain; RNase_Ire1, endoribonuclease domain of *Saccharomyces cerevisiae* Ire1, a multifunctional protein essential for the endoplasmic reticulum unfolded protein response (UPR); PKc: catalytic domain of protein kinases.
