# Supplementary material for: A Non-canonical RNA Silencing Pathway Promotes mRNA Degradation in Basal Fungi
Source: PLoS Genet. 2015 Apr 13;11(4):e1005168. doi: 10.1371/journal.pgen.1005168 (PMC4395119; doi:10.1371/journal.pgen.1005168)
Supplement: S4 Table — (DOCX) [file pgen.1005168.s018.docx]

| **Gene** | **Name** | **Sequence^1^** |
| --- | --- | --- |
| **136157** | F1 | 5’ ACATCCATTTGTTTACAGCAGCAAAG 3’ |
|  | F2 | 5’ GTAACATGCACTCAGAAGCACATGGCGTCA 3’ |
|  | F3 | 5’ AT*CTGCAGAGATCT*CCGAGGACTTTGTCAGAAATCTCCAGCA 3’ |
|  | R1 | 5’ GGAACCACTCACTAAAACCTCCTA 3’ |
|  | R2 | 5’ ACATGCTGAGCCTGATGTTTAGAAAGCACGA 3’ |
|  | R3 | 5’ AT*CTGCAGAGATCT*TTCTCCGTGTATAGAAACACCACTGGCAAG 3’ |
| **110239** | F4 | 5’CAGTTTCTGGATAGTGGAAGCAGCAG 3’ |
|  | F5 | 5’ GGGTAACGAGATGATACAATACTGGCAGGA |
|  | F6 | 5’ TA*GGATCC*ATGAGGTCACGAACATGTACAATGCATC 3’ |
|  | R4 | 5’ ATACATTGGCATCCTCCACATCCGA 3’ |
|  | R5 | 5’ GATAATCCAGCCATGTATTCCTGGTAGCTC 3’ |
|  | R6 | 5’ AA*GGATCC*AATCAAGGTGCAGGAACCCAAGCGTCA 3’ |
| **77996** | F7 | 5’ TGCTTGTGACTCATATGTGTGCTG 3’ |
|  | F8 | 5’ CCTGTAGGTGAGGAACGCTGTATCGCTT 3’ |
|  | F9-pyrG | 5’**CGATAGCATGGCCAGTGTAC**AAATGACGCTCAACATCTCGCATTCGACA 3’ |
|  | R7 | 5’ TGCTCTTTGCTGATGATTCTCCAG 3’ |
|  | R8 | 5’ GTCCAAGGCAGCTTCCTCGTTGTAGAC 3’ |
|  | R9-pyrG | 5’ **CAAGTACCAATGCTGAGGCA**AACTGTCTTCGGCCTTGTTGGTGCTG 3’ |
| **80729 (*r3b2*)** | F10 | 5’ AGACGTCTTGATCGCTGTGG 3’ |
|  | F11 | 5´GGGGG*TCTAGA*TTGCTGAGGGAACATTGGACCTGGATGAGC 3´ |
|  | R11 | 5´ GGGGG*CTCGAG*CGTACTACTGCAAAACATAGCCTGAGCGGG 3´ |
|  | F12 | 5’ CCCCC*GGATCC*AGATGATTGAGCTTGTCACCA 3’ |
|  | R12 | 5’ GGGGG*GGATCC*CTAGGATCAGGTGCATGGCG 3’ |
|  | F13 | 5’ GGGGG*CTCGAG*TTGCTGAGGGAACATTGGACCTGGATGAGC 3’ |
|  | MutFow | 5’ GAGGCCAAGTTCAACTACATTGCAGCGTCTGCAGTGC 3’ |
|  | MutRev | 5’ AGACGCTGCAATGTAGTTGAACTTGGCCTCTGCCACCTC 3’ |
| ***pyrG*** | pyrG10 | 5´ GGCAGAAGGGAGGAGGCACACG 3´ |
|  | pyrg-F2 | 5’ GGCAAGTAACACCACATTCAGAGC 3’ |
|  | pyrg-R2 | 5’ ATCCCACCAGAAGGAGTACATGG 3’ |
|  | F-pyrG | 5’ TGCCTCAGCATTGGTACTTG 3’ |
|  | R-pyrG | 5’ GTACACTGGCCATGCTATCG 3’ |
| **Others** | leuA-Sal | 5’ AAGAAT*GTCGAC*ATACTCTAACTACATCAAATGC |
|  | peuK-Sal | 5’ TCCCCAA*GTCGAC*TTGGGCCCAAGCTTTCAAATG 3’ |
|  | carRP-F1 | 5’ AATC*GCATGCCCGGG*CGCATTGTAGATAAACTCG 3’ |
|  | carRP-R1 | 5’ TCGCT*GGTACCCGGG*CATGTGTAACAGTGCATTGG 3’ |
|  | carRP-R2 | 5’ AGGAC*CTGCAG*CCATATTGAGTCATCCTGC 3’ |

**Table S4. Oligonucleotides used for cloning and functional analysis of the candidate RNase genes.**

^1^Underlined and italic nucleotides indicate restriction sites used for cloning. Bold nucleotides correspond to *pyrG* sequences used for fusion PCR. Underlined nucleotides indicate changes relative to the wild type sequence.
