# Supplementary material for: A Non-canonical RNA Silencing Pathway Promotes mRNA Degradation in Basal Fungi
Source: PLoS Genet. 2015 Apr 13;11(4):e1005168. doi: 10.1371/journal.pgen.1005168 (PMC4395119; doi:10.1371/journal.pgen.1005168)
Supplement: S1 Supporting Information — Detailed description of plasmid construction and generation of knock-out mutants. (DOC) [file pgen.1005168.s022.doc]

**S1 Supporting Information**

**Supplementary Materials and Methods**

**Plasmid construction and site-directed mutagenesis**

Plasmid pMAT1294 contains a 3.44 kb genomic fragment that includes the *r3b2* coding region and adjacent sequences (874 bp upstream and 1029 bp downstream). It was amplified from genomic DNA using oligonucleotides F11 and R11 (S4 Table), which include restriction sites for *Xba*I and *Xho*I, respectively, and cloned into pBluescript vector. Plasmid pMAT767 harbors a 3.4 kb fragment containing the complete 136157 gene and adjacent regions. This fragment was PCR-amplified using primer pairs F2 and R2 (S4 Table) and cloned into pBluescript vector. Plasmid pMAT762 contains a 2.5 kb genomic fragment that includes the entire 110239 locus and flanking regions. It was generated by PCR-amplification using primers F5 and R5 (S4 Table) and cloned into pBluescript vector.

Plasmid pMAT1298 was constructed to disrupt the *r3b2* gene and contains the *M. circinelloides* *pyrG* gene (which complements the uracil auxotrophy of the MU402 strain) flanked by *r3b2* sequences. In brief, plasmid pMAT1294 was inverse PCR-amplified using oligonucleotides F12 and R12 (S4 Table), which contain *BamH*I restriction sites. These primers amplified a 5.1 kb fragment that includes the vector sequence flanked by 1.07 kb and 1.05 kb of sequences adjacent to *r3b2*. The *BamH*I-digested PCR product was ligated with a 3.4 kb *BamH*I fragment isolated from pEMP1, which includes a wild type allele of the *M. circinelloides* *pyrG* gene, to produce pMAT1298. A 5.5 kb replacement fragment was released from plasmid pMAT1298 by *Xba*I and *Xho*I double digestion and introduced into MU402 by transformation (see S8 Fig.). Plasmid pMAT768 was constructed to disrupt the 136157 gene following a similar strategy, using plasmid pMAT767 as a template for inverse PCR amplification with primers F3 and R3 (S4 Table), which contain *Bgl*II restriction sites. The amplified 5.1 kb fragment was *Bgl*II digested and ligated with the 3.4 kb *pyrG* fragment to give plasmid pMAT768. A 5.5 kb replacement fragment harboring the *pyrG* gene flanked by 1.1 kb and 1.0 kb of sequences adjacent to 136157 was released from plasmid pMAT768 by *Pvu*II digestion, amplified with primers F2 and R2 (S4 Table) and introduced into MU402 by transformation (see S9 Fig.). Similarly, plasmid pMAT763 was constructed to disrupt the 110239 gene, using plasmid pMAT762 as a template for inverse PCR amplification with primers F6 and R6 (S4 Table), which include *BamH*I restriction sites. The 4.6 kb amplified fragment was digested with *BamH*I and ligated with the 3.4 kb *pyrG* fragment. A 5.0 kb replacement fragment harboring the *pyrG* gene flanked by 0.8 kb of upstream and downstream sequences of 110239 was released from plasmid pMAT763 by *Pvu*II digestion, amplified with primers F5 and R5 (S4 Table) and introduced into MU402 by transformation (see S10 Fig.). Plasmid pMAT770 was constructed to disrupt the 77996 gene. It contains a 4.0 kb fragment that includes the *pyrG* gene flanked by upstream and downstream sequences of the 77996 gene. It was constructed by fusion PCR using primer pairs F7/R9-pyrG (S4 Table), and F9-pyrG/R7 (S4 Table) to amplified 1.0 kb of upstream and downstream sequences of the 77996 locus, respectively. Those fragments were used in a fusion PCR together with a 2 kb *pyrG* fragment amplified from plasmid pEMP1 using primers F-pyrG and R-pyrG (S4 Table). The 4 kb fusion fragment was amplified with internal primers F8 and R8 (S4 Table) and cloned into pGEM-T easy to give plasmid pMAT770. The 4.0 kb replacement fragment was released from plasmid pMAT770 by *Pvu*II digestion, amplified with primers F8 and R8 and introduced into MU402 by transformation (see S10 Fig.).

Plasmid pMA771 was used to complement the *r3b2^-^* mutation. To construct this plasmid, the 3.44 kb fragment of pMAT1294 that includes the *r3b2* coding region and adjacent sequences was amplified with primers F13 and R11 (S4 Table), which contain *Xho*I restriction sites, and the *Xho*I-digested fragment was ligated to a 10 kb *Sal*I-digested fragment that had been amplified from pMAT1253 [12] using primers leuA-Sal and peuk-Sal (S4 Table). The obtained plasmid, pMAT771, contains the wild type allele of the *r3b2* gene, a silencing-reporter *carB* transgene expressing a hairpin RNA and the *leuA* gene as selectable marker.

Plasmid pMAT772 contains the *r3b2** mutant allele, the hairpin RNA-expressing *carB* transgene and the *leuA* marker. The *r3b2** mutant allele, which contains the missense substitutions H49A, G55A and E56A affecting conserved residues of the R3B2 RNase III-like domain, was generated by site-directed mutagenesis on pMAT1294 DNA by overlap extension using fusion PCR [50]. Briefly, two overlapping PCR fragments of *r3b2* were amplified in a first round of PCR using the inner primers MutRev and MutFow (S4 Table), which correspond to the sequences to be mutated, and primers F13 and R11 (S4 Table) corresponding to upstream and downstream sequences to the sequences to be mutated (outer primers). In a second round of PCR, the mutagenized *r3b2** region was generated with only outer primers and the first-round PCR products as templates. The authenticity of the sequence of the *r3b2** mutant allele was confirmed by sequencing. The 3.44 kb fragment containing the *r3b2** mutant allele was cloned into the 10 kb *Sal*I-digested pMAT1253 fragment to give plasmid pMAT772.

Plasmid pMAT787 was constructed to integrate the *r3b2* gene into the *carRP* locus. To construct this plasmid, the 3.4 kb *Xho*I fragment isolated from pMAT771 was cloned into pMAT1476 [51], a plasmid that includes the *M. circinelloides* *leuA* marker gene flanked by upstream and downstream sequences of the *carRP* gene. The *Xho*I site of pMAT1476 allows cloning of *r3b2* gene just downstream of *leuA* and, thus, keeping *r3b2* within the sequences flanked by the *carRP* upstream and downstream regions. A 9.8 kb integration fragment was released from plasmid pMAT787 by *Cfr*9I digestion, amplified with primers carRP-F1 and carRP-R1 (S4 Table) and introduced into the MU412 (*r3b2^-^* *leu^-^*) strain by transformation (see S12 Fig.). Plasmid pMAT788 was constructed in a similar way, except that the *r3b2* fragment was excised from plasmid pMAT772 and contains point mutations in conserved residues of the R3B2 RNase III-like domain (*r3b2** mutant allele). The 9.8 kb integration fragment containing the *r3b2** mutant allele flanked by the *carRP* upstream and downstream regions was isolated from pMAT788 and used to transform the *r3b2^-^* strain as indicated above (see S12 Fig.).

**Generation of knockout mutants for the RNase genes**

Knockout vectors pMAT1298, pMAT768, pMAT763 and pMAT766 were designed to disrupt *r3b2*, 136157, 110239 and 77996 genes, respectively. Restriction fragments from each plasmid containing the *pyrG* gene flanked by sufficient sequences of the candidate RNase genes to allow homologous recombination were used to transform the MU402 strain (Ura^-^, Leu^-^). Ura^+^ transformants were grown in selective medium for several vegetative cycles to increase the proportion of transformed nuclei, as primary transformants are heterokaryons due to the presence of several nuclei in the protoplasts. Homokaryotic transformants were PCR analyzed to distinguish homologous from ectopic integrations, using primer pairs pyrG10 and F10 (*r3b2* gene) (S8B Fig.), pyrg-R2 and F1 (136157 gene), pyrg-F2 and R4 (110239 gene) and pyrg-F2 and R7 (77996 gene) (S4 Table) to identify homologous integration events in the corresponding loci. Disruption of each gene was confirmed by Southern analysis (S8-S10 Figs.). Digested DNA from each transformant was hybridized with specific probes that recognized both wild type and mutant alleles but could discriminate between them (probes a, b, d and e, S8-S10 Figs.) or corresponded to a DNA region deleted in the knockout vector (probe c, S9 Fig.). Several analyzed transformants showed the expected fragments derived from the corresponding mutant alleles and the absence of the wild type ones, confirming that those mutants have successfully replaced the wild type allele (S8C and S9B Figs.). Those mutants were named MU412 (*r3b2*) and MU450 and MU451 (136157). The mutant allele in MU412 has replaced 1.3 kb of the *r3b2* coding region by the *pyrG* gene. The mutant alleles in MU450 and MU451 have completely eliminated the 136157 coding region and replaced it by the *pyrG* gene. All those mutant strains were considered null mutants. Transformants harboring mutant alleles were also obtained for 110239 and 77996 genes. However, it was impossible to derive homokaryotic null mutants from those transformants, since all of them maintained wild type nuclei even after incubation for more than ten vegetative cycles in selective medium to increase the proportion of transformed nuclei, as indicated by the presence of both mutant and wild type fragments in Southern analysis (S10C and S10D Figs.).

**Supplementary References**

50. Ho SN, Hunt HD, Horton RM, Pullen JK, Pease LR. Site-directed mutagenesis by overlap extension using the polymerase chain reaction. Gene 1989;77: 51-59.

51. Rodríguez-Frómeta RA, Gutiérrez A, Torres-Martínez S, Garre V. Malic enzyme activity is not the only bottleneck for lipid accumulation in the oleaginous fungus *Mucor circinelloides*. Appl Microbiol Biotechnol. 2013; 97: 3063-3072.
